# Controlling the breakup of toroidal liquid films on solid surfaces

**DOI:** 10.1038/s41598-021-87549-5

**Published:** 2021-04-14

**Authors:** Andrew M. J. Edwards, Élfego Ruiz-Gutiérrez, Michael I. Newton, Glen McHale, Gary G. Wells, Rodrigo Ledesma-Aguilar, Carl V. Brown

**Affiliations:** 1 SOFT Group, School of Science and Technology, Nottingham Trent University, Clifton Lane, Nottingham NG11 8NS UK; 2 Institute for Multiscale Thermofluids, School of Engineering, University of Edinburgh, The King’s Buildings, Mayfield Road, Edinburgh, EH9 3FB UK; 3 Smart Materials and Surfaces Laboratory, Department of Mathematics, Physics and Electrical Engineering, Northumbria University, Ellison Place, Newcastle upon Tyne, NE1 8ST UK

**Keywords:** Condensed-matter physics, Fluid dynamics

## Abstract

The breakup of a slender filament of liquid driven by surface tension is a classical fluid dynamics stability problem that is important in many situations where fine droplets are required. When the filament is resting on a flat solid surface which imposes wetting conditions the subtle interplay with the fluid dynamics makes the instability pathways and mode selection difficult to predict. Here, we show how controlling the static and dynamic wetting of a surface can lead to repeatable switching between a toroidal film of an electrically insulating liquid and patterns of droplets of well-defined dimensions confined to a ring geometry. Mode selection between instability pathways to these different final states is achieved by dielectrophoresis forces selectively polarising the dipoles at the solid-liquid interface and so changing both the mobility of the contact line and the partial wetting of the topologically distinct liquid domains. Our results provide insights into the wetting and stability of shaped liquid filaments in simple and complex geometries relevant to applications ranging from printing to digital microfluidic devices.

## Introduction

Drops and bubbles adjust their shape to minimise the surface energy, for instance, becoming spherical when suspended^1^. However, drops and bubbles can exist, at least temporarily, in geometries of a higher free energy. Such geometries are inherently unstable, driven by surface tension to revert back to the more energetically favourable shape. A free liquid jet, for example, while cylindrical at the nozzle outlet, breaks into spherical droplets by virtue of the Plateau–Rayleigh (P–R) instability^2,3^, as volume for volume, spheres have smaller surface area than cylinders. For a thin inviscid liquid cylinder, with radius, *r*, and length, *L*, small periodic perturbations with wavelength, $\lambda > 2\pi r$ on the surface of the liquid become amplified over time^4^. However, not all perturbations grow equally. The wavelength of the fastest growing perturbation is given by the universal relation, $\lambda _{\text {c}} = 9.02r$ and so the mode of breakup is given by the simple geometric relation $n = L/\lambda _c$^3,5^.

Free floating toroidal shaped droplets and bubbles are another example of a nonminimal energy shape which regularly occurs in nature. These can be found when droplets impact on superhydrophobic surfaces^6^, be created by rapid oscillation of the surface, by high rising speeds^7–9^ or by shaped magnetic fields^10^. Free floating toroidal shaped droplets and bubbles can breakup through the capillary driven P–R instability, with the most unstable breakup mode being related to the geometry of the torus^11–13^. Their unique topology allows an additional shrinking instability which is not possible in a cylinder. This acts to close the inner hole of the toroidal liquid filament, thus re-forming to a single sphere, and is denoted as an $n = 0$ mode. Therefore, during the energy minimisation of a toroidal shaped droplet there exists a competition between the P–R instability and the shrinking instability, and so the shape and the topology add complexity to understanding the mode of breakup^14^.

Such complexity is further increased when a small droplet of a partially-wetting liquid is placed on a solid surface, where the balance of the solid–vapour, $\gamma _{\text {sv}}$ solid–liquid, $\gamma _{\text {sl}}$ and liquid vapour, $\gamma$ surface tensions determines the static wettability of the surface and forces the liquid to take the shape of a spherical cap shaped droplet intersecting the solid with Young’s equilibrium angle, $\cos \theta _{\text {e}} = (\gamma _{\text {sv}}-\gamma _{\text {sl}})/\gamma$^15^. When out of equilibrium, surface tension acts as a restoring force to minimise the overall energy of the system^1^. This leads to the motion of the contact line, controlled by both the equilibrium angle and the localised slip, $\ell$, on the surface. For a higher energy geometry, such as a toroidal liquid filament on a substrate^16^, hydrodynamic instabilities and static and dynamic wettability act to minimise the overall energy of the system^17,18^. However, the subtle interplay between each of these effects and the timescales over which they act makes understanding the pathway to minimisation, and therefore predicting the outcome, complicated^17,19,20^.

Here, we report how localised controllable surface wettability is able to produce and control the dewetting of toroidal liquid films into toroidal liquid filaments and their eventual break up into droplets. We use dielectrowetting to produce the initial ring-shaped filament of electrically insulating liquid on the normally liquid repellent surface. We study the dynamics and resultant breakup modes of toroidal films after the dielectrowetting actuation is abruptly switched off. We use Fourier series and linear stability analysis (LSA) to elucidate the subtle role that both static and dynamic wettability play in the liquid shape evolution. Lastly, by control over the degree of surface wettability through our dielectrowetting method, we elucidate selection of the pathway and mode during the evolution of a toroidal liquid filament.

## Results

### Pathways to energy minimisation in toroidal insulating liquid films

**Figure 1:**
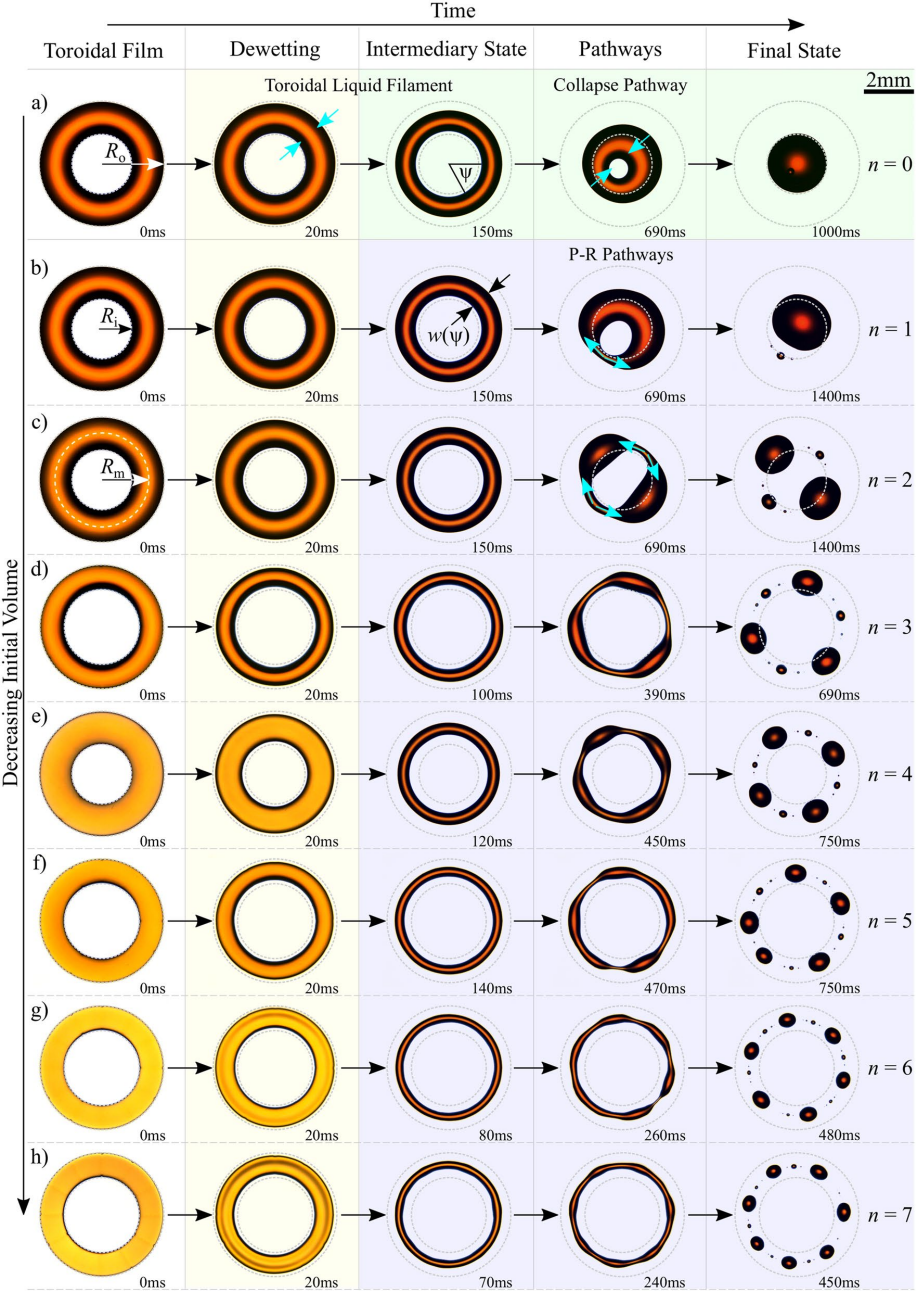
Experimental results on the pathways to energy minimisation for initial spread toroidal-shaped liquid films having different deposited volumes. (**a**) $2.8 \pm 0.2 \,  \upmu \hbox {L}$. (**b**) $2.5 \pm 0.2  \,  \upmu \hbox {L}$. (**c**) $1.7 \pm 0.2  \,  \upmu \hbox {L}$. (**d**) $0.9 \pm 0.2  \,  \upmu \hbox {L}$. (**e**) $0.8 \pm 0.2  \,  \upmu \hbox {L}$. (**f**) $0.5 \pm 0.1  \,  \upmu \hbox {L}$. (**g**) $0.4 \pm 0.1  \,  \upmu \hbox {L}$. (**h**) $0.3 \pm 0.1  \,  \upmu \hbox {L}$. Images of the initial toroidal liquid films are shown in the left hand column that were produced using ring-shaped regions of electric field controlled variable wettability (regions subsequently marked by grey dashed circles for comparison). The evolution of the liquid shape is shown in each case in subsequent columns at different times after a sudden quench of the electric field to zero. Blue arrows show the direction of movement of the contact line and azimuthal flows. Images edited for brightness and contrast using Fiji (version 1.52p, https://imagej.net/Fiji).

To form our initial toroidal spread film of electrically insulating liquid we use non-uniform electric fields generated between sub-surface electrodes to polarise the electric dipoles within the liquid, immediately adjacent to the solid-liquid interface. This dielectrowetting^21^ increases the effective local surface wettability, and provides the unique ability to impose a pattern of voltage controlled partial, to full, wettability onto a normally non-wetting solid surface. This allows a variety of desired shaped contact and wetting areas to be defined by the electrode geometry, and here we have created liquid wetting rings. We form thin spread toroidal films of an electrically insulating dielectric liquid, trimethylolpropane triglycidyl ether (TMP-TG-E) (liquid vapour surface tension $\gamma = 43.27 \pm 0.25 \, \hbox {mN/m}$, and dynamic viscosity $\eta = 210 \pm 20 \, \hbox {mPa.s}$ @ $20^{\circ }\hbox {C}$^22^), from initial spherical cap shaped droplets (see Supplementary Movie M1) by patterning our surfaces with a series of inter-digitated electrodes arranged in a concentric ring array, capped with a thin planarising dielectric layer and a hydrophobic surface coating (See “Methods” section). Upon application of an A.C. voltage, *V*, between electrodes a non-uniform electric field localised at the surface is created^23,24^. Deposited droplets respond by spreading over the solid until they cover an area such that the overall energy, having surface tension and electrostatic components, is minimised. The electrostatic energy component is purely dielectric for an electrically insulating liquid such as TMP-TG-E used in this work, and is associated with the polarisation of bound charge in induced dipoles in the liquid in the absence of free charges. The induced increase in wetting area is accompanied by a reversible reduction in the solid-liquid contact angle, $\theta$, with increasing *V*. Above a threshold voltage, $V_{\text {th}} \approx 300 \, \hbox {V}$, the liquid spreads completely over the solid patch above the electrodes to create a toroidal film that remains held in place due to the electrostatic actuation, see the left hand column of Fig. 1. It is important to note that the ability to form such a reversible, spread, ring-shaped liquid film on a solid flat surface is unique to our dielectrowetting approach. This would not be possible using an alternative Electrowetting on Dielectric (EWOD)^25,26^ approach which suffers from contact angle saturation^27,28^, since in EWOD the limited extent to which the contact angle can be reduced would therefore prevent the liquid from spreading down to the initial spread film state that we can achieve using dielectrowetting.

By suddenly setting the voltage *V* between electrodes to zero, we rapidly quench the electric field, and hence the patterning of increased surface wettability is abruptly and completely removed, introducing a sudden change in the free energy of the system. This forces the shape of the toroidal liquid film to evolve, driven by surface tension forces, to adopt the shape that minimises the total free energy (see Fig. 1 and Supplementary Movie M2). Initially, this occurs by a rapid dewetting process occurring locally at both the inner and outer contact lines with a speed, *U* driven by the deviation from equilibrium of the contact angles^22,29^. During this rim dewetting phase the liquid shape evolves from a spread toroidal film to take the form of a toroidal liquid filament, keeping the memory of the initial patterning of the liquid film. We define two geometric measurements while the toroidal liquid filament remains unbroken; $R_{\text {m}}$, which is the mean of the inner and outer radii, $R_{\text {i}}$ and $R_{\text {o}}$, respectively, and $w(\psi )=R_{\text {o}}(\psi )-R_{\text {i}}(\psi )$ which is the width of the torus as a function of the azimuth angle $\psi$ (see Fig. 1). To simplify our discussion, we also define the average width of the torus, denoted by ${\bar{w}}$, and an aspect ratio as $AR \equiv R_{\text {m}} / {\bar{w}}$. For the filament, the reduced width *w* is now dependent on the liquid volume and the instantaneous contact angle $\theta$. The toroidal liquid filament continues to dynamically evolve in shape to minimise the surface free energy. The global minimum energy state corresponds to a single droplet, thereby losing memory of the original film topology with its central hole, see the $n = 0$ mode in Fig. 1a. However, with decreasing liquid volume, we observe a number of disconnected droplets distributed around the original ring geometry, for example see the $n = 1$ and $n = 2$ modes in Fig. 1b and Fig. 1c, respectively. These latter droplet states result from the Plateau–Rayleigh (P–R) instability and have a higher total surface energy for a fixed volume than the single droplet state.

In general terms, the evolution of the liquid shape and selection of pathway is a competition between the timescales of dewetting and P–R breakup. The timescale of dewetting is given by, $t_{\text {dewetting}} \sim \Delta x / U^*$, where $\Delta x$ is the dewetting lengthscale, $U^* = \gamma \theta _{\text {e}}^3 / \eta \ln (w / \xi )$ is the characteristic velocity and $\xi$ is a microscopic cut-off length scale^22^. While the timescale of the mode with maximum growth rate for P–R instability in a toroidal liquid filament can be given by $t_{n^*} \sim 8 R_{\text {m}}/U^{*\,} AR$, and central collapse, $t_{n = 0} \sim 3 R_{\text {m}} AR / U^*$ (see Supplementary Information for details). Therefore, the initial pathway to minimisation for all final states is a rapid shrinking of the torus width from both contact lines, $t_{\text {dewetting}} \approx 100\,{\text {ms}}$ resulting in the intermediary state. In this state the aspect ratio for the torus is increased, thereby decreasing the timescale of each P–R mode. If $t_{n^*} > t_{n = 0}$, then the droplet evolves by the radial movement of both contact lines towards the center resulting in a single on-axis droplet, termed $n = 0$ mode. If $t_{n^*} < t_{n = 0}$, P–R instability breakup now dominates the minimisation pathway and the fastest growing P–R mode drives azimuthal flows forcing “pinch-off” resulting in a string of disconnected droplets distributed around the original ring geometry. Therefore, for a fixed volume and surface wettability, the geometrical quantity of the aspect ratio at the intermediary state directly controls the two competing timescales. Then the selection of energy minimisation pathway is the one which locally reduces the free energy the quickest and whilst not necessarily being a global minimisation of energy (see Fig. 1 and Supplementary Movie M2).

**Figure 2:**
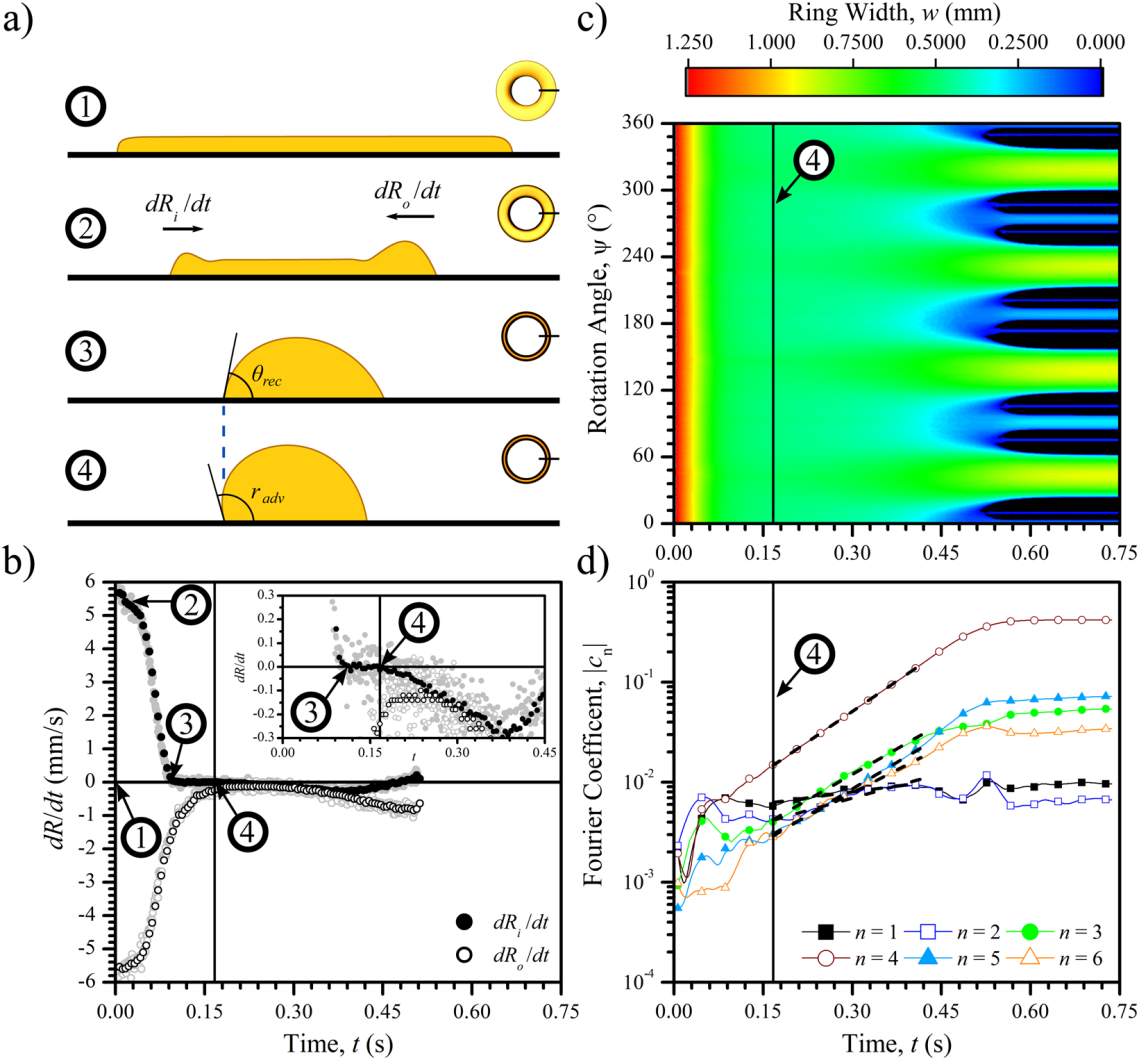
Characterisation of the evolution of an $n = 4$ breakup induced by quenching the electric field for a droplet of deposited volume, $0.8 \pm 0.2  \,  \upmu \hbox {L}$. (**a**) Schematic representation of the cross section of the liquid ring marked by the solid line. Not to scale. (**b**) Plot of inner, ${\mathrm {d}}R_{\text {i}}/{\mathrm {d}}t$ (solid circles), and outer, ${\mathrm {d}}R_{\text {o}}/{\mathrm {d}}t$ (open circles), edge velocities as a function of time. The grey shaded points are the raw experimental data and the black points show the low-pass filtered data. Inset shows a zoom in on the intermediary phase. (**c**) Contour plot of the toroidal liquid filament width *w* with time *t* as a function of the azimuthal angle $\psi$. (**d**) Time evolution of the first six coefficients from a Fourier analysis of $w\left( \psi ,t\right)$. The vertical solid line marks the end of the intermediary phase at $t=0.16 \, \hbox {s}$. Dashed lines show linear fit to data at subsequent times. Images edited for brightness and contrast using Fiji (version 1.52p, https://imagej.net/Fiji).

To understand the pathways of minimisation, we quantify how a toroidal film evolves as a function of time. The results of these measurements for an $n = 4$ mode breakup with a schematic cross section of a segment of the toroidal film are shown in Fig. 2 (See Supplementary Movie M3). At the initial state, the liquid is shaped as a film of even thickness covering the electrode area (stage 1). As the electric field is quenched the dewetting phase begins and both inner and outer contact lines recede forming two capillary rims that move towards each other (stage 2). For clarity, we have exaggerated the flatness of the film and relative height of the formed capillary rims for this volume in Fig. 2a. We plot the velocities of the inner, ${\mathrm {d}}R_{\text {i}}/{\mathrm {d}}t$, and outer, ${\mathrm {d}}R_{\text {o}}/{\mathrm {d}}t$, edge velocities as a function of time (see Fig. 2b). When the two rims merge, the contact lines slow down and the contact angles increase. Eventually, the inner contact line stops moving and the contact angle raises to the receding contact angle (stage 3). However, the outer contact line is still decelerating, and by conservation of mass, the fluid in the outer section is drawn inwards, which further increases the inner contact angle up to the advancing contact angle (stage 4). This event defines the intermediary state, shown by the vertical solid line, which corresponds to when ${\mathrm {d}}R_{\text {i}}/{\mathrm {d}}t = 0$ is no longer constant and starts to become negative as the inner radius commences to decrease and experimentally is found by the onset of motion of the inner contact line (${\mathrm {d}}R_{\text {i}}/{\mathrm {d}}t = 0$ and ${\mathrm {d}}R_{\text {o}}/{\mathrm {d}}t \approx 0$ simultaneously, see inset in Fig. 2b). The end of the dewetting phase results in a shape of an unstable equilibrium, i.e., the toroidal liquid filament.

Figure 2c shows the instantaneous width of the toroidal liquid filament using a colour scale, as a function of time on the horizontal axis and azimuthal angle on the vertical axis. At the start there is a rapid decay of the width at all angles, arising from the initial dewetting phase ($t < 0.11 \, \hbox {s}$). After the dewetting phase, the toroidal liquid filament enters the intermediary phase ($0.11 \, \hbox {s}< t < 0.16 \, \hbox {s}$), in which the toroidal liquid filament is nearly uniform along the entire circumference and the liquid filament edges are approximately stationary. At the end of the intermediary phase minor sinusoidal perturbations develop along the circumference of the inner and outer edges of the filament due to P–R instability. For continuity, these distortions must have a wavelength which is an integer fraction of the overall contact line circumference $n \lambda = 2\pi R$^13^.

To visualise the time evolution of each mode, we carry out a Fourier series analysis on the width of the toroidal liquid filament as a function of the azimuthal angle, $w\left( \psi ,t\right)$. Thus, $c_n$ corresponds to the weight of the *n*-th mode of the Fourier series $w\left( \psi ,t\right) = \sum _n c_n \left( t\right) e^{-in\psi }$. Fig. 2d shows the Fourier coefficients for the first six modes during the time evolution of the toroidal liquid filament. Close examination immediately after the intermediate state ($t > 0.16 \, \hbox {s}$) reveals that several modes are growing simultaneously and exponentially with time following the relation $|c_n| \propto e^{\omega t}$, where $\omega$ is the growth rate. Our analysis shows that for this experiment, whilst at the end of the dewetting phase the coefficients of $|c_1|$ and $|c_4|$ modes are similar and each coefficient grows with time, it is the $|c_4|$ coefficient which grows fastest, resulting in the $n = 4$ final state. Therefore, using Fourier analysis we are able to quantify the growth rate of each of the coefficients, and, without loss of generality, find the fastest growing mode that results in the final pattern.

### Mathematical model

We construct a mathematical model to analyse the behaviour during breakup of a relaxing toroidal liquid filament. In the absence of electric fields, the dynamics is governed by the competition between capillary forces and viscous dissipation. During the intermediate stage, the liquid flows radially or azimuthally leading to the $n = 0$ mode collapse or the $n \ge 1$ mode P–R pathways, respectively. In both cases, the characteristic lengthscale is proportional to mean of the inner and outer radii, $R_{\text {m}}$. Considering that the typical thickness of the fluid is much smaller than $R_{\text {m}}$, we model the breakup dynamics of the toroidal liquid filament using the thin-film equation in the long wavelength approximation^17,30^,

1 $$\begin{aligned} 3 \eta \partial _t h + \gamma \nabla \cdot \left[ h^2 (h + 3 \ell ) \nabla \kappa \right] = 0, \end{aligned}$$

where *h* is the local thickness of the dielectric liquid, $\kappa$ is the local mean curvature of the interface, and $\nabla$ is the gradient operator in cylindrical coordinates $(R,\Psi ,z)$, with *R* and *z* being coordinates parallel and perpendicular to the wall, respectively. We note that Eq. (1) is strictly valid in the limit of small contact angles, where the flow is approximately parallel to the solid wall. To model the contact angles observed in the experiments^31^, we retain the full expression for the curvature: $\kappa = -\nabla \cdot \hat{{\varvec{n}}}/2$, where $\hat{{\varvec{n}}}$ corresponds to the unit normal vector to the interface. Such an approximation does not capture the details of the flow close to the contact line, but retains the main effect of driving and dissipative forces. In Eq. (1), it has been assumed that the Navier boundary condition is satisfied at the liquid-solid interface for the velocity of the flow in the radial direction, $u_R(z = 0)$^32^,

2 $$\begin{aligned} u_R(z = 0) = \ell \, \partial _z u_R(z = 0), \end{aligned}$$

which defines the contact line slip length $\ell$^32^ mobility parameter (see Supplementary Information for more details).

**Figure 3:**
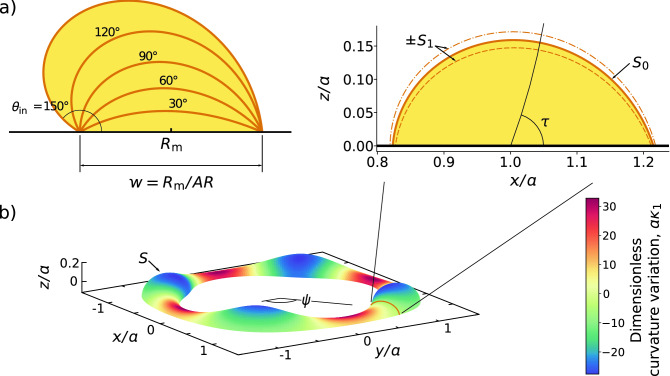
Results of the mathematical model. (**a**) Equilibrium solution as constant curvature solutions prescribed by the positions of the contact lines fixing the aspect ratio to $AR = 3.72$. A contact angle can also be prescribed by adjusting the value of the curvature $\kappa _0$. (**b**) Theoretically generated shape of a perturbed state of positive growth at $\psi = 0$ ($\epsilon = 0.008$ for visibility). (**c**) Surface plot of the perturbed interface ($\epsilon = 0.064$). In this illustration $n = 4$, $\theta _{\text {out}} = 72^\circ$ and $\ell / R_{\text {m}} = 0.16$.

To take advantage of the symmetry of the system and model high contact angles ($\sim 90^\circ$), we parameterise the interface profile in toroidal coordinates as

3 $$\begin{aligned} h = \frac{a \sin \tau }{\cosh S - \cos \tau }, \end{aligned}$$

where *a* defines unit of length of the coordinate system and is given by $a = (R_{\text {i}}R_{\text {o}})^{1/2}$ at the beginning of the intermediate phase. The surface $S = S(\tau , \psi , t)$, corresponds to the liquid-vapour interface parameterised by the two angles $\tau$ and $\psi$ (see Fig. 3). For example, constant *S* results in a torus of constant minor radius. Eq. (3) has the advantage that *h* becomes single-valued, and thus, alleviated from the singularities of contact angles above $90^\circ$ that Eq. (1) produces.

Prior to the intermediate phase, the outer contact line slows down tending to form a static receding contact angle. However, the inner contact line has stopped its motion and remains immobile for some time while building up to form a static advancing contact angle. This difference of contact angles is due to hysteresis and allows the toroidal liquid filament to come close to an equilibrium state that we denote by $S_0$. From this point, we carry out a Linear Stability Analysis (LSA) to solve Eq. (1) by performing a single mode perturbation,

4 $$\begin{aligned} S(\tau , \psi , t) = S_0(\tau ) + \epsilon S_1(\tau ) \cos n \psi \, e^{\omega t}. \end{aligned}$$

In Eq. (4), the last term corresponds to the Fourier mode of amplitude $\epsilon$ and exponential growth rate $\omega$, as observed in the experiments. In the same way, the curvature is expressed to linear order in $\epsilon$, $\kappa = \kappa _0 + \epsilon \kappa _1$, where $\kappa _1$ corresponds to the variation from equilibrium.

We begin by finding the equilibrium shape; this corresponds to an interface of constant curvature, $\kappa [S_0] = \kappa _0$, and is uniquely determined by the position of the two contact lines or, equivalently, $R_{\text {m}}$ and *AR*. Therefore, we vary $\kappa _0$ to set the outer contact angle to match the experimental observations (see Fig. 3a).

After obtaining $S_0$, we proceed to solve Eq. (1) to first order in $\epsilon$ by substitution of Eq. (4)^20,30^. The resulting equation corresponds to an eigensystem, where $\omega$ corresponds to the eigenvalue and $S_1$ the eigenfunction. The growth rate of a Fourier mode is found by the largest eigenvalue for a given *n*. For consistency, we enforce Eq. (2) as the boundary conditions for $S_1$. The correspondence is established by calculating the velocity of the flow at the contact lines, i.e.,

5 $$\begin{aligned} u_R(z = 0) = \pm \, \epsilon \, a \, \omega \, S_1 \tanh ^{\pm 1}(S_0/2) \, \cos n \psi \, e^{\omega t}\big |_{\tau = \pi , 0} \end{aligned}$$

where the choice of signs corresponds to $\tau = \pi$ and $\tau = 0$, respectively. In this way we are able to model the friction forces at the contact line and consequently the dynamics of the contact angle (see Fig. 3b). For slip lengths of magnitude $\ell \gg R_{\text {m}}$, the change in the contact angle becomes negligible as the friction forces are reduced. For $\ell = 0$, in contrast, the contact lines become pinned and thus unable to move.

We proceed to find the shape and growth rate of the Fourier modes. To illustrate our results, in Fig. 3b, we present an example of an $n = 4$ mode, mirroring the experimental situation showing in Fig. 2a. The gradient of the change in pressure, $- \epsilon \gamma \nabla \kappa _1$, drives the flow inside the toroidal liquid filament, therefore, if the $\kappa _1$ is negative in the lobe-shaped regions and positive in the thin ones, the perturbation has a positive growth rate.

From the model, we find that there are three key parameters which control the stability of the Fourier modes, a geometric parameter, *AR*, a static wetting parameter, $\theta _{\text {out}}$ and dynamic wetting parameter, $\ell$. From the experimental images we are able to determine the values of $\theta _{\text {out}}$ and *AR* for each of our experiments (see “Methods”) meaning the only free parameter to match the model to the experiments is the slip length, $\ell$. To match the model with the experiments, we proceed to find the value for $\ell$ that fits the experimental results when the electric field is quenched.

### Pathways in uniform surface wettability

**Figure 4:**
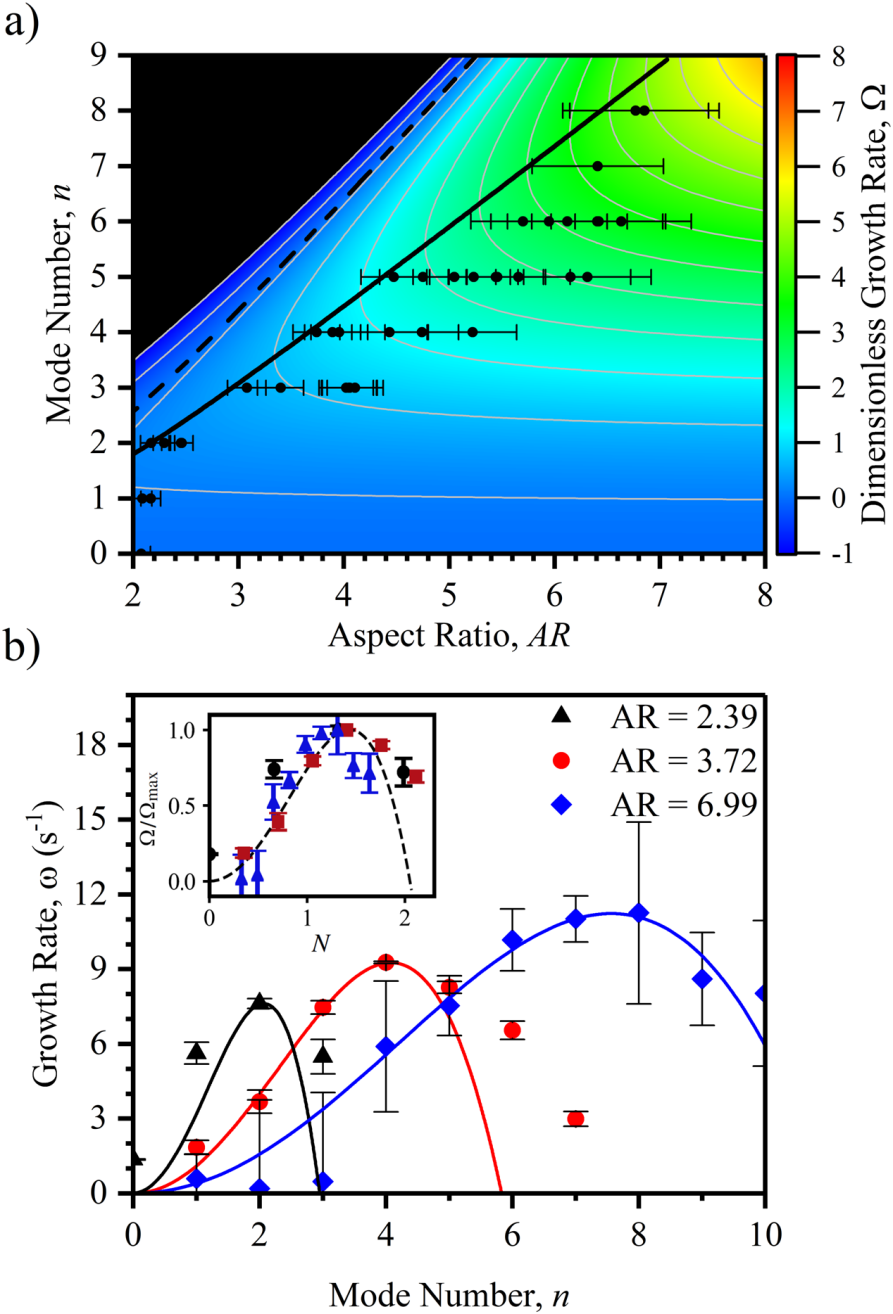
Comparison between experimental observations and LSA predictions for quenching of the electric field. (**a**) Phase diagram of the modal growth rates as a function of the intermediary stage aspect ratio. The experimental data is shown as black circles. The maximum growth-rate for a given *AR* is represented by the solid line, the dashed line corresponds to marginal stability $\left( \omega = 0\right)$. Contour lines show equal growth rate. (**b**) Comparison between experimentally measured modal growth rates at the onset of the Plateau–Rayleigh phase (symbols) and the theoretical prediction (solid lines) for three different experimental aspect ratios. Black—AR = 2.39 ($1.7 \pm 0.2  \,  \upmu \hbox {L}$). Red—AR = 3.72 ($0.8 \pm 0.2  \,  \upmu \hbox {L}$). Blue—AR = 6.99 ($0.3 \pm 0.1  \,  \upmu \hbox {L}$). The value of the contact angle at the outer contact line is used as $\theta _{\text {out}} = {72}{^{\circ }}$, the fitted slip-length is $\ell /R_{\text {m}}=0.16\pm 0.03$. Inset shows the collapse of the data on to the single master curve.

We now compare our mathematical model to the experimental results on the dewetting of toroidal insulating liquid films into toroidal liquid filaments and their eventual break up into droplets which is initiated by quenching the electric field (i.e. setting $V=0$) to restore the original intrinsic uniform surface wettability, see Figs. 1 and 2a (also Supplementary Movie M2). Figure 4a shows the phase diagram of the theoretical modal growth rates in dimensionless form, $\Omega \equiv 3 \omega \eta /\gamma a$, as a function of the aspect ratio over which the experimental data has been plotted (black circles in Fig. 4a). For our experimental data, the aspect ratio for each experiment is measured at the end of the dewetting phase, while the mode number is the mode of the final breakup pattern for that experiment. The error bars show the error in measurement of *AR* due to the error in locating the exact end of the dewetting phase, as for higher aspect ratios the P–R instability begins to emerge during dewetting (See Supplementary Movie M2). The dashed and solid lines show the predicted marginal stability limit and the maximum growth rate at a given aspect ratio, respectively. The value of the contact angle at the outer contact line $\theta _{\text {out}}$ comes directly from the side view of experimental measurements (${72}{^{\circ }} \pm {1}{^{\circ }}$) and the only fitting parameter used to match the theoretical predictions to the experimental data is the slip length, $\ell$. It should be noted that, since the mode $n = 0$ is always present, the aspect ratio decreases during the breakup state. This implies that the growth rates are dynamically changing, and the mode number for maximum growth-rate reduces. Therefore, the maximum growth-rate curve is shifted slightly above the experimental results. However, we observe a strong correlation between the maximum predicted growth rate and the final modal outcome of each experiment across a wide range of measured intermediary state aspect ratios.

A more direct comparison between the mathematical model and the experimental results can be made from analysing the measured and predicted growth rates of each mode. Figure 4b shows a comparison between the experimentally measured growth rates (symbols) from Fourier analysis plotted against the theoretical predictions (solid lines) for three different aspect ratios. Although the values that *n* can acquire are strictly integers, by analytical continuity, we are able to explore the behaviour at intermediate values. It is clear that changing the aspect ratio changes the growth rates of the different modes. A low aspect ratio represents a liquid torus of large tubular radius, and higher *n* modes increase the surface energy and therefore $\omega < 0$. On the other hand, higher aspect ratios represent narrow tori and thus higher order modes become energetically favourable with a positive growth-rate. Increasing the aspect ratio resembles a linear stripe described by the Plateau–Rayleigh instability^33^. Thus, for a superposition of Fourier modes of random amplitude, the mode with highest growth rate is more likely to dominate the breakup of the toroidal liquid filament. Overall, the theoretical predictions are in good agreement with the experimental data.

From the inset of Fig. 4b, it can be observed that the modal growth rate is well captured by a fourth order polynomial (dashed curve in the inset) in *n*, where odd powers in *n* do not appear since the Fourier modes are invariant upon a change in sign of *n*. Moreover, at high aspect ratios and low contact line mobility, $\omega (n = 0) \approx 0$, and so the independent term can be neglected. This implies that the polynomial form can be expressed in terms of two independent coefficients, for instance, a constant of proportionality, and the maximum modal growth for a given aspect ratio, $n_{\text {max}} = \{ n \,|\, \omega (n_{\text {max}}) \ge \omega , AR = {\text {const.}} \}$. Furthermore, as can be seen in Fig. 4a, the maximum modal growth follows a straight line at high aspect ratios, therefore a transformation can be made to reduce the parameter space into a master curve (see inset in Fig. 4b),

6 $$\begin{aligned} \Omega (n, AR, \theta _{\text {out}}, \ell ) \propto N^2 (2 N_{\text {max}}^2 - N^2), \end{aligned}$$

where $N = n / (AR - \beta )$, is the transformation to collapse into a master curve and $N_{\text {max}} = n_{\text {max}} / (AR - \beta )$ corresponds to the maximum modal growth. Both, $\beta$ and $N_{\text {max}}$ depend on the outer contact angle $\theta _{\text {out}}$ and slip length, $\ell$. In the Supplementary Information, we define approximate expressions to the numerical results obtained for $\beta$ and $N_{\text {max}}$.

**Figure 5:**
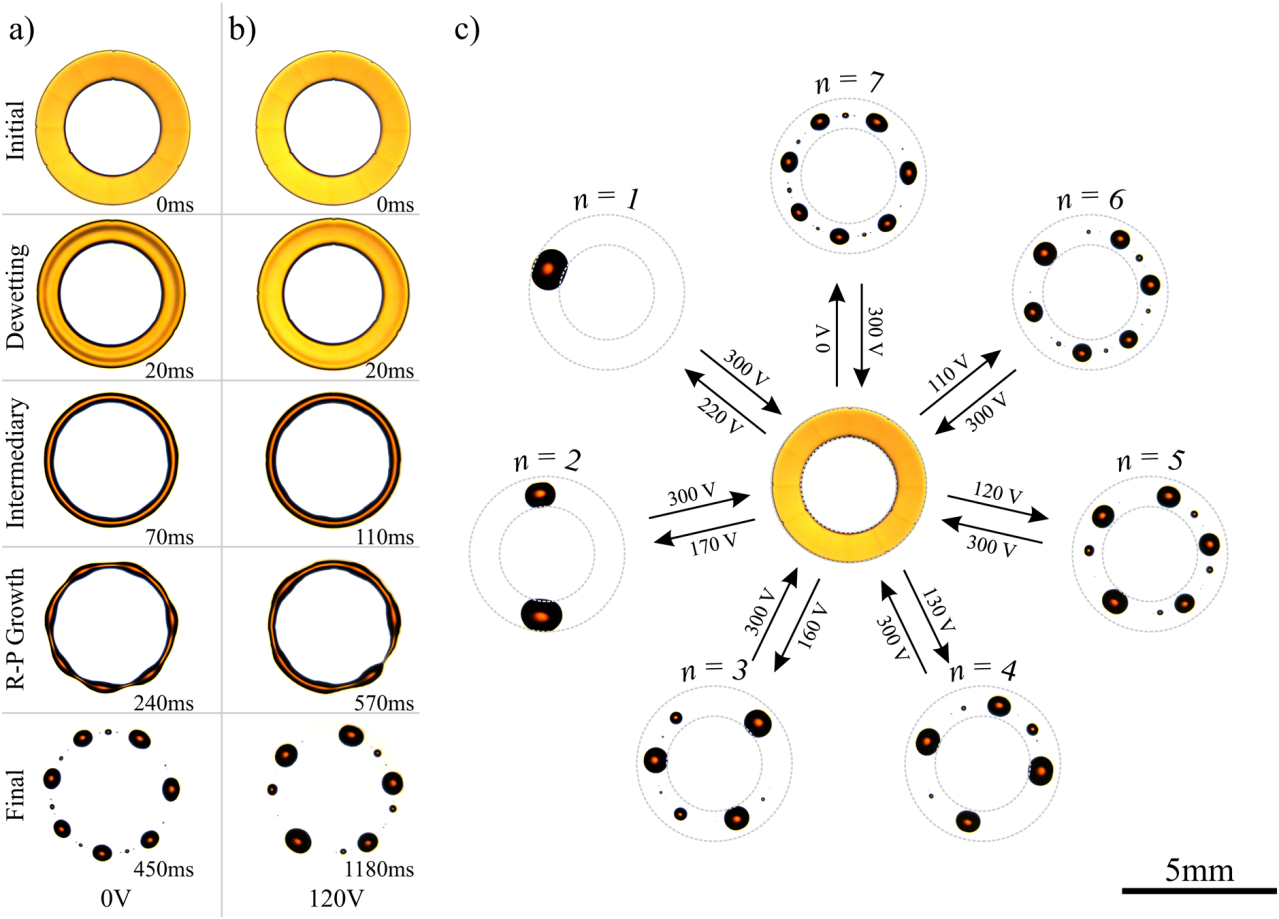
Electric field modulation of the Plateau–Rayleigh instability pathway for a fixed volume of $0.3 \pm 0.1  \,  \upmu \hbox {L}$ by application of a retraction voltage $V_{\text {R}}$. (**a**) Time sequence images of an evolving toroidal liquid film at $V_{\text {R}} = 0 \, \hbox {V}$. (**b**) Time sequence experimental images of the same evolving toroidal liquid film at $V_{\text {R}} = 120 \, \hbox {V}$. (**c**) Formation of lower order modes, $n = 6$, 5, 4, 3, 2 and 1 from the same initial toroidal film by localised controllable surface wettability ($V_{\text {R}} \ne 0$). Images edited for brightness and contrast using Fiji (version 1.52p, https://imagej.net/Fiji).

### Pathway selection by electric field patterned wettability

We now quantify how a toroidal film evolves as a function of time throughout the shape evolution following the sudden reduction in the voltage value, in contrast to complete voltage removal, at $t = 0$. Rather than quenching the electric field ($V = 0 \, \hbox {V}$), we instead switch the applied voltage to a non-zero retraction voltage, $V = V_{\text {R}}$, which is lower than $V_{\text {th}}$, thereby retaining a geometrically patterned wettability. This allows for the elucidation of the role of static and dynamic wettability on the pathways, with the degree of wettability controlled by $V_{\text {R}}$. The voltage controlled wettability, denoted by $\theta (V_{\text {R}}) \le \theta _{\text {e}}$, is increased within the patterned ring area defined by the electrodes, in comparison with the lower wettability on the surrounding outer and enclosed inner solid areas^34^. In this situation, the toroidal film driven by surface tension forces still evolves via dewetting followed by P–R breakup. However, the dominant P–R breakup mode *n* is now reduced, by a number determined by the value of $V_{\text {R}}$, in comparison with the identical quenching experiment (i.e. when $V_{\text {R}}=0,{\text {V}}$). To demonstrate this effect we choose a very low liquid volume, $0.3 \pm 0.1  \,  \upmu \hbox {L}$, to provide an intermediary state that has a high aspect ratio, and which results in a high final P–R mode. Figure  5 shows how exactly the same initial toroidal film that results in an $n = 7$ mode when $V_{\text {R}} = 0\,{\text {V}}$, switches to an $n = 5$ mode when $V_{\text {R}} = 120\,{\text {V}}$ (see Fig. 5a,b). To study the effect of $V_{\text {R}}$, we re-spread the droplets back to the initial toroidal film using $V > V_{\text {th}}$ and repeatedly switch to increasing retraction voltages i.e. final increased surface wettability (see Fig. 5c and Supplementary Movie M4). Figure 5c shows that the dominant P–R instability mode decreases as the surface wettability increases. Here the $n = 0$ pathway is excluded because the voltage maintains a higher wettability within the annular area of the ring, compared to the area without electrodes within the centre of the ring. Measurements of the aspect ratio at the intermediary state show that the aspect ratio decreases with increasing voltage, following an approximately linear relationship for $V_{\text {R}} \ge 70\,{\text {V}}$ (see Supplementary Fig. 4). However, we find that the modification of the aspect ratio at the intermediary state alone is insufficient to fully explain the different final breakup patterns.

**Figure 6:**
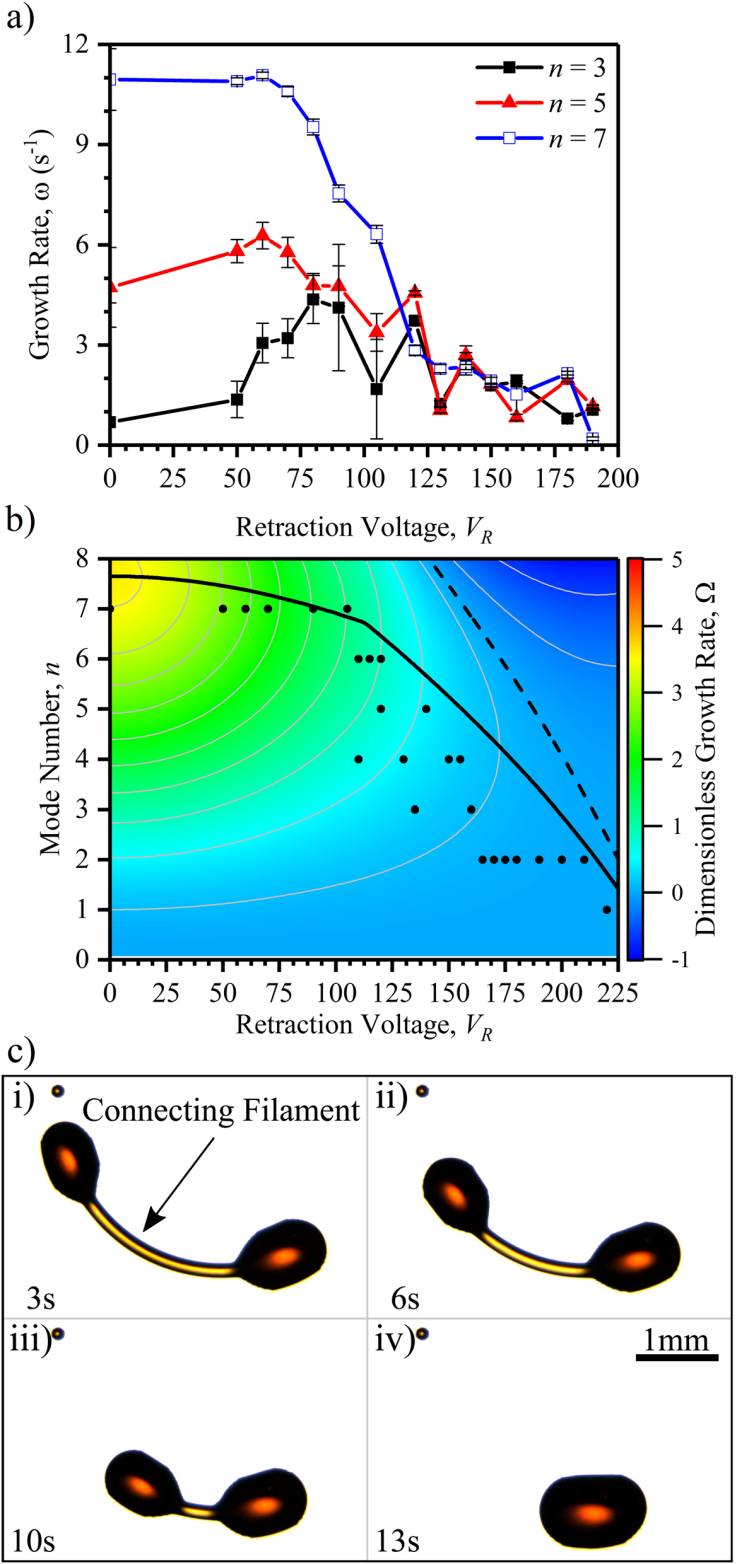
Growth rates of the voltage modulated experiments ($V_{\text {R}} \ne 0$). (**a**) Growth rates of various Fourier modes as a function of applied retraction voltage obtained from experimental fitting. (**b**) Phase diagram of the growth rates of possible modes as a function of the applied voltage, using $\ell _0 / R_{\text {m}} = 0.16 \pm 0.03$, $b' = 3.42 \times 10^{-5} {\mathrm {V}}^{-2}  \,  \upmu {\mathrm {m}}^{-1}$ and $\gamma = 43.27\,{\text {mN}}/{\text {m}}$. Contour lines show equal growth rate. (**c**) Time sequence images showing the stabilisation of a thin connecting liquid filament between main droplets by localised electric field reduction of contact line mobility ($V_{\text {R}} = 180 \, \hbox {V}$). Leading to joining of the main droplets and suppression of satellite droplets produced by the Plateau–Rayleigh instability. Images edited for brightness and contrast using Fiji (version 1.52p, https://imagej.net/Fiji).

We use the Fourier analysis technique to examine how the electric field patterning of the wettability of the surface determines the final P–R breakup pattern demonstrated in Fig. 5. Fig. 6a shows the experimentally measured growth rates of three Fourier modes, $n = 3$, 5 and 7 for a range of retraction voltages. It is clear that, as the retraction voltage increases, and hence the wettability of the surface increases, the growth rate of each mode is modified. For the $n = 7$ mode, the growth rate rapidly declines above $70\,{\text {V}}$, to about $14 \%$ of its initial value at $160\,{\text {V}}$. The growth rate of the $n = 5$ mode remains predominantly unaffected until around $120\,{\mathrm {V}}$ when it begins to decrease. The $n = 3$ mode growth rate initially shows a rise around $60\,{\text {V}}$ which is biased by the geometry of the underlying electrode structure, nonetheless, the growth rate of this mode decreases back towards its original value (with scatter) at higher voltages. Therefore, at low voltages higher order modes are the fastest growing, dominating the breakup pattern. As the retraction voltage increases, the stronger suppression of higher order modes enables lower order modes to dominate, with growth rates becoming increasingly equivalent at higher voltages.

In the dielectrowetting driven electric field patterning of the static wettability, the contact angle is explicitly linked to the applied voltage between electrodes^21,34^. Therefore, switching to non-zero retraction voltages causes the toroidal film to relax during the dewetting phase to a contact angle lower than the equilibrium angle. The voltage-dependent angle is modelled as $\cos \theta (V) = \cos \theta _0 + f_{\text {el}}/\gamma$, where $f_{\text {el}} = \Delta \varepsilon V^2 / 2 \delta$ is the dielecrophoretic force per unit length, where $\left( \Delta \varepsilon = \varepsilon _{\text {l}}-\varepsilon _{\text {v}}\right)$ is the difference between the liquid and vapour permittivities, $\varepsilon _{\text {l}}$ and $\varepsilon _{\text {v}}$ respectively, and $\delta$ is the penetration depth^21^. For the present experiments, $\Delta \varepsilon /2 \gamma \delta = 1 \times 10^{-5} \, {\hbox {V}}^{-2}$ (see Supplementary Information). This effect explains how the aspect ratio decreases with increasing voltage. For a given volume, a higher voltage promotes wettability and a lower contact angle, which leads to a wider and shallower cross-section of the toroidal liquid filament with a lower aspect ratio. From our Linear Stability Analysis, we find that the lower contact angle, representing increased wettability, dominates the selection of the breakup mode more than aspect ratio alone as lower contact angles are more stable against the P–R instability.

The motion of the contact line is affected by the retraction voltage as, during initial dewetting and relaxation, the contact line moves orthogonally to the underlying electrode geometry through the spatially varying electric field. At the edges of the electrode stripes the electric field is at peak intensity which creates stagnation points and introduces voltage dependent pinning^21,35^. Taking into account the exponential evolution of the Plateau–Rayleigh instability, the pinning force is proportional to the velocity of the contact line and to the dielectrophoretic force (see Supplementary Information). Therefore, the balance of stresses that give rise to the boundary condition of Eq. (2), now includes a term that depends on $V_{\text {R}}$ and restricts the mobility of the contact line,

7 $$\begin{aligned} \left[ \frac{\eta }{\ell } + b f_{\text {el}}(V_{\text {R}}) \right] u_R = \eta \partial _z u_R \end{aligned}$$

where *b* is a constant of proportionality to be found by the experimental results. Equivalently, we can define an effective slip length of the contact line, $\ell _{\text {eff}} = \ell _{\text {eff}}(V_{\text {R}})$, defined by the reciprocal relation, $\ell _{\text {eff}}^{-1} := \ell ^{-1} + b' V_{\text {R}}^2$, where $b' \approx 3.68 \Delta \epsilon / 2 \gamma \delta R_{\text {m}} = 3.42 \times 10^{-5} {\mathrm {V}}^{-2}  \,  \upmu {\mathrm {m}}^{-1}$. At $V_{\text {R}} = 0$ the slip length is unaltered, $\ell _{\text {eff}}(0) = \ell$. As the retraction voltage $V_{\text {R}}$ is increased, $\ell _{\text {eff}}$ decreases, with $\ell _{\text {eff}} \rightarrow 0$ as $V_{\text {R}} \rightarrow \infty$.

We introduce the voltage dependent behaviour of the contact angle and slip length (mobility) of the contact line into the mathematical model to use the voltage as the control parameter and compare against our experimental results. Figure 6b shows the phase diagram of the theoretical voltage controlled model growth rates in dimensionless form over which the experimental data has been plotted (black circles in Fig. 6b). The observed scatter of the experimental data occurs from the quantization of the final mode value within each experiment and is expected given the nearly equivalent growth rates of competing modes at higher voltages (see Fig. 6a). The experimental data points show a close agreement with the maximum growth rate curve (solid line).

We note here that the effect of reducing contact line mobility at higher voltages greatly inhibits movement of the contact line in the radial direction (see Fig. 6c and Supplementary Movie M4). Fig. 6c (i) shows two main droplets produced by the P–R instability during a voltage modulated experiment ($V_{\text {R}} = 180 \, \hbox {V}$) connected by a thin liquid filament. Over time the connecting filament decreases in length (see Fig. 6c (ii) and (iii)) and the two main droplets join together resulting in a single final droplet (see Fig. 6c (iv)). The thin connecting liquid filaments between the main droplets are therefore stabilised against breakup and the preferred energy minimisation method is axial retraction. Therefore, our patterning of wettability is able suppress the formation of secondary and tertiary droplets between the main droplets produced by the P–R instability.

## Discussion

In this work, we have shown how patterning of surface wettability by a non-contact electric field method is able to generate and select the pathway of energy minimisation for toroidal dielectric liquid films. We have studied the time evolution of the minimisation in detail, using Fourier analysis to elucidate how the complex interplay between geometry, and static and dynamic wettability results in the selection of pathway to the final state. The experimental observations of the pathways of evolution have been modelled using a linear stability analysis of a thin-film finite-slip model which uses only two free parameters, the contact angle and slip length, predicting with accuracy all aspects of the time evolution. We further use this understanding to interpret the selection of the pathway to the final states by the electric field control i.e., a combination of altering static and dynamic surface wettability by contact angle and contact line mobility reduction respectively.

Experimentally, we have focused on the dynamics of toroidal liquid films and pathways to mode-selected droplet states. However, the experiments, theoretical analysis and the control of the most unstable mode of P–R breakup can be applied to geometries beyond the toroidal geometry and topology explored here. Our non-contact method based on an underlying electrode structure defines the area of increased wettability when the voltage is applied. This allows the creation of liquid films with a variety of initial topologies such as liquid disks, where all of the liquid is simply connected, rings, where the liquid film has a central hole or more complex shapes including disconnected and non-simply connected volumes of liquid. This enables the study of the interactions of different instabilities and pathways to final states. Therefore, our approach bridges the gap between understanding the behaviour of more complex shapes and the simplest case of a liquid stripe^36^ as exemplified by our findings for the pathways to different final toroidal droplet states arising from an initial toroidal shaped film. Moreover, our findings are relevant to other surface bound unstable systems such as those formed during spinodal dewetting^37^, long liquid filaments^38,39^ and pulsed laser induced dewetting (PLiD) of metallic films^18,40^.

Lastly, the ability to select the pathway of the P–R instability has applications in the opto-electronics industry providing re-configurable optical elements with complex geometries including being able to form lenslet shapes of uniform size without secondary droplets^41,42^. The ability to actively suppress secondary and tertiary droplet formation by modifying the stability of high order modes on interconnecting liquid filaments can prevent loss of material in lab on a chip device^43^.

## Methods

### Device geometry and fabrication details

Annular arrays of interdigitated co-planar parallel micro-stripe indium tin oxide (ITO) electrodes were fabricated on the solid surface using standard photolithographic techniques. The electrode linewidths, of $20 \,  \,  \upmu \hbox {m}$, are equal to the electrode gaps. To allow access to a large range of modes two annular patterns were used in the experiments, one with dimensions $R_{\text {i}}=1.22 \, \hbox {mm}$, $R_{\text {o}}= 2.54 \, \hbox {mm}$ and $w_0 = 1.32 \, \hbox {mm}$ and one with dimensions $R_{\text {i}}=1.54 \, \hbox {mm}$, $R_{\text {o}}= 2.54 \, \hbox {mm}$ and $w_0 = 1 \, \hbox {mm}$. The surface and the electrodes were coated with a SU-8 dielectric layer (Microchem Corp., thickness $1 \,  \,  \upmu \hbox {m}$, dielectric constant 3.2) to planarise the surface and to prevent any charge injection into the electrically insulating liquid. To promote retraction of the TMP-TG-E when the applied voltage is removed a thin Teflon AF mixed in a 0.5% by weight solution with its solvent, was applied. Substrates were dip-coated in the solution allowed to dry at room temperature before baking at ${155}{^{\circ }} \hbox {C}$ for 20 min to cure. The surface roughness of the coated samples was measured using an Veeco Dektak 6M surface profilometer across the active electrode areas. The measurements show that the surfaces on the small scale have an arithmetic mean deviation surface roughness of $26 \pm 9 \, \hbox {nm}$ for the Teflon AF surface.

### Equipment and settings

The application of voltage *V* to alternate electrodes in the interdigitated electrode array (with interposed electrodes at earth potential) was performed by an Agilent 33500B waveform generator providing a 10 KHz sine wave to a PZD700A (Trek Inc.) amplifier, which multiplies the input signal 100x. Root Mean Square values of the A.C. voltage *V* are given in this report. The integrity and instantaneous amplitude of the output waveform was monitored using an DSO6014A (Agilent) oscilloscope, and the applied voltage was measured using a 34410A (Agilent) digital voltmeter where the error is found to be $\pm 0.5 \, \hbox {V}$. The timescale of switching between different voltages was measured in situ to be $<50  \,  \upmu \hbox {s}$. Images were captured from both the side and top during the experiments. Top images were captured using an EO-13122C (Edmund Optics) fitted with a x4 objective at 100 FPS. For the data shown in Fig. 2 we use a HHC x4 camera (Mega Speed Corporation) fitted with a x4 objective lens at 1000 FPS. Side images for contact angle analysis were captured using an HHC x4 camera (Mega Speed Corporation) fitted with a x5 objective lens up to 1000 FPS. To improve image contrast for the image analysis the TMP-TG-E was dyed using Sudan Orange II (CAS number: 3118-97-6) at a concentration of 0.1% by wt.

### Image analysis

Images are edited for brightness and contrast in a pre-processing step using Fiji (version 1.52p, https://imagej.net/Fiji). For top images, the positions of the inner and outer edges as a function of the azimuthal angle of the dewetting toroidal liquid filament are measured using a bespoke developed MatLAB program (Academic Licence, Version R2019a, MathWorks, https://uk.mathworks.com/products/matlab.html) using the image processing toolbox. The average position of the inner, $R_{\text {i}}$ and outer, $R_{\text {o}}$ contact lines is determined using a circle fitting algorithm applied to the measured respective edge data. From each of these measurements we are able to compute the velocity of each contact line, and measure the width of the toroidal liquid filament as a function of time and azimuthal angle. Side images are analysed using a bespoke MatLAB program (Academic Licence, Version R2019a, MathWorks, https://uk.mathworks.com/products/matlab.html) using the image processing toolbox, which enables measurement of the apparent outer contact angle $\theta _{\text {out}}$ and apparent equilibrium angle $\theta _{\text {e}}$. The contact angle is calculated by fitting a tangent to points above a user determined baseline, this tangent is then extrapolated to find the respective apparent contact angle at the solid substrate. We estimate deposited droplet volume from the top view images by averaging the measurement of the long and short axis of each resultant droplet, which is used in conjunction with the equilibrium angle, $\theta _{\text {e}} = 80 \pm {2}{^{\circ }}$, to calculate individual resultant droplet volume. These individual droplet volumes are then summed to give the initial volume of the deposited droplet, we consider satellite droplet volume negligible.

## Supplementary Information

Supplementary Legends.

Supplementary Information.

Supplementary Movie M1.

Supplementary Movie M2.

Supplementary Movie M3.

Supplementary Movie M4.
