# Supplementary material for: Controlling the breakup of toroidal liquid films on solid surfaces
Source: Sci Rep. 2021 Apr 14;11:8120. doi: 10.1038/s41598-021-87549-5 (PMC8046813; doi:10.1038/s41598-021-87549-5)
Supplement: Supplementary file 1 — Supplementary Legends. [file 41598_2021_87549_MOESM1_ESM.docx]

{Supplementary Information}

Supplementary Movie M1

The formation of a thin toroidal 1m of a dielectric liquid, trimethylolpropane triglycidyl ether (TMP-TG-E), from spherical cap shaped droplets when a voltage is applied to inter-digital electrodes arranged in a concentric ring array.

Supplementary Movie M2

The voltage is removed from thin toroidal films of dielectric liquid, rapidly quenching the electric field. Each film has a different liquid volume, and hence aspect ratio (AR), leading to the different Plateau-Rayleigh instability pathways that exhibit different order break-up modes.

Supplementary Movie M3

The time evolution of the n = 4 break-up mode of a thin toroidal 1m (with zero retraction voltage). This high-speed recording was used for the analysis shown in figure 2 of the manuscript.

Supplementary Movie M4

The eect of applying varying retraction voltages to a high aspect ratio toroidal droplet. Different order break-up modes, n = 6, 5, 4, 3, 2 and 1 are produced from exactly the same initial toroidal 1m by localised controllable surface wettability.

Supplementary Information file F1: Theory

Details the analytical model that we have developed and used to analyse the stability of toroidal droplets.
