# Supplementary material for: Controlling the breakup of toroidal liquid films on solid surfaces
Source: Sci Rep. 2021 Apr 14;11:8120. doi: 10.1038/s41598-021-87549-5 (PMC8046813; doi:10.1038/s41598-021-87549-5)
Supplement: Supplementary file 2 — Supplementary Information. [file 41598_2021_87549_MOESM2_ESM.pdf]

Supplementary Information
Controlling the breakup of toroidal liquid films on solid surfaces

Andrew M. J. Edwards[1,+], Élfego Ruiz-Gutiérrez[2,3,+], Michael I. Newton[1], Glen McHale[2,3], Gary G. Wells[2,3], Rodrigo Ledesma-Aguilar[2,3,*], and Carl V. Brown[1,*]

[1]SOFT Group, School of Science and Technology, Nottingham Trent University, Clifton Lane, Nottingham NG11 8NS, UK.
[2]Institute for Multiscale Thermofluids, School of Engineering, University of Edinburgh, The King's Buildings, Mayfield Road, Edinburgh EH9 3FB, UK.
[3]Smart Materials and Surfaces Laboratory, Department of Mathematics, Physics and Electrical Engineering, Northumbria University, Ellison Place, Newcastle upon Tyne NE1 8ST, UK.
[*]email:carl.brown@ntu.ac.uk, rodrigo.ledesma@ed.ac.uk

Timescales

During the dewetting stage (see Fig. S1), both contact lines recede forming a capillary rim at the inner and outer sides of the liquid film (2). As the capillary rims merge, the contact lines slow down and both contact angles approach the receding contact angle (3). At some point, the inner contact line stops moving, however, the outer contact line has not reached equilibrium yet and the outer contact line continues its motion. Due to volume conservation, the liquid from the outer part of the toroidal liquid filament is pushed to the centre increasing the inner contact angle. This occurs until the contact angle reaches the advancing contact angle (4). If the difference between the contact angles of the inner and outer sides is within the contact angle hysteresis created by the surface, the toroidal liquid filament can arrive close to an unstable equilibrium state, i.e., the toroidal liquid filament.

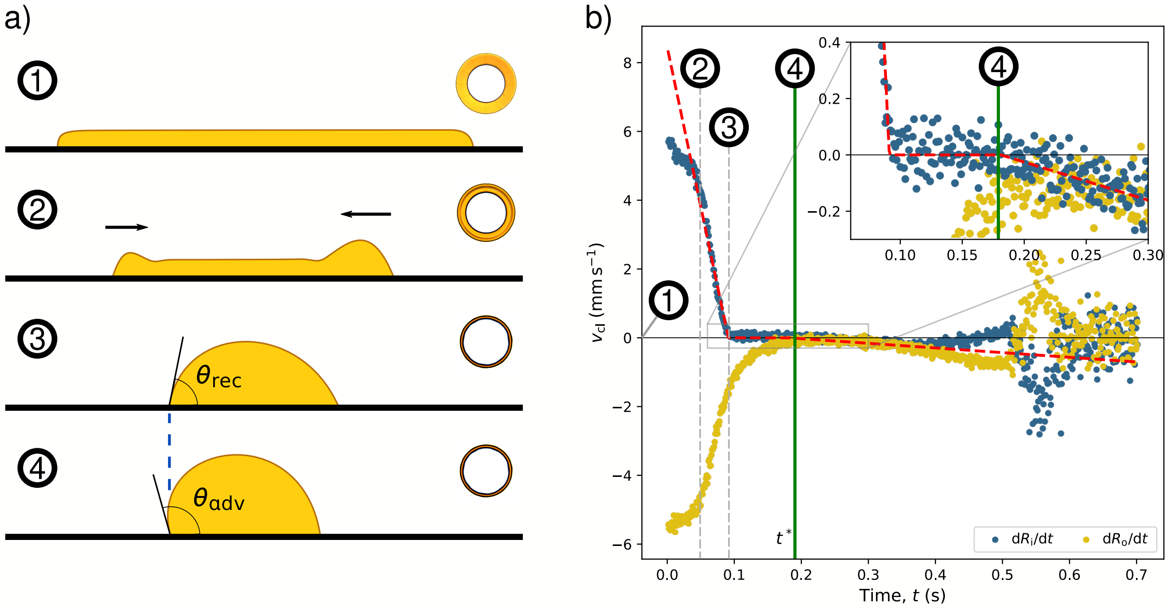

Supplementary Figure S1: The dewetting phase and arrival to the temporal equilibrium. (a) Schematic representation of the cross section of the toroidal liquid filament. (b) Plot of velocity of the inner and outer contact lines as a function of time.

The velocity of the contact lines during the initial dewetting stage is given by the balance between the capillary forces and the viscous friction caused by the flows. The flows near the contact line distort the shape of the interface, causing the contact angle to deviate while the capillary forces act to revert the angle to equilibrium. This results in the Cox–Voinov relation, which for an inviscid surrounding phase and a wide range of contact angles (below $3\pi/4$) it can be written as [1]

$$\theta^3 - \theta_\mathrm{e}^3 \approx \frac{\eta}{\gamma} v_\mathrm{cl} \log \frac{\bar{w}}{\xi} \tag{1}$$

where v_cl is the velocity of the contact line, θ and θ_e correspond to the dynamic and equilibrium contact angle and ξ is a microscopic cut-off length scale. Solving for v_cl, we find

$$v_\mathrm{cl} = -U^* \left(1 - \frac{\theta^3}{\theta_\mathrm{e}^3}\right), \tag{2}$$

where we define $U^* \equiv \gamma\theta_\mathrm{e}^3/\eta \ln(\bar{w}/\xi)$ as the characteristic velocity. During the initial dewetting stage, the difference between the dynamic and the equilibrium contact angles is highest, with $\theta < \theta_\mathrm{e}$. Therefore, neglecting the last term in the brackets of Eq. (2), an approximate timescale for dewetting can be given by

$$t_\mathrm{dewetting} \sim \frac{\Delta x}{U^*} \approx 0.1\,\mathrm{s}, \tag{3}$$

where Δx is the distance that the contact line moves during the dewetting stage and the value of $U^* \simeq 5\,\mathrm{mm\,s^{-1}}$ is obtained from the experimental measurements (See Supplementary Figure S1).

Once the dewetting stage is over, the toroidal liquid filament can relax and move towards the centre coalescing to a single droplet, or a string of off-axis droplets depending on the dominant pathway, radial collapse or a PR breakup, respectively. As the toroidal liquid filament slowly arrives to the intermediate stage, contact angle hysteresis allows the inner and outer contact angles to adjust, the internal eddies start to dissipate and the pressure is homogenised.

For the radial collapse take place, the difference between the inner and outer contact angles must surpass contact angle hysteresis, and therefore, the net restoring force rises from its imbalance. According to González, et al. [2], the inner and outer contact angles must satisfy,

$$(2AR-1)\left[2AR-(2AR+1)^2\,\mathrm{acoth}2AR\right]\tan\theta_\mathrm{in} + (2AR+1)\left[2AR-(2AR-1)^2\,\mathrm{acoth}2AR\right]\tan\theta_\mathrm{out} = 0. \tag{4}$$

for the toroidal liquid filament to form. Due to contact angle hysteresis, we can take the equilibrium contact angle, θ_e, to be the average between the two angles, i.e., $\theta_\mathrm{e} = (\theta_\mathrm{in} + \theta_\mathrm{out})/2$. Then, Eq. (4) can be written as,

$$4AR\left[(1-4AR^2)\,\mathrm{acoth}2AR + 2AR\right]\cos^2\theta_\mathrm{e} + 2\left[(1-4AR^2)\,\mathrm{acoth}2AR - 2AR\right](\theta_\mathrm{in}-\theta_\mathrm{out}) + O(\theta_\mathrm{in}-\theta_\mathrm{out})^2 = 0. \tag{5}$$

Further more, taking the Laurent series for $1/AR$ around 0, we obtain

$$-4AR(\theta_\mathrm{in}-\theta_\mathrm{out}) + \frac{2}{3}\sin 2\theta_\mathrm{e} + \frac{\theta_\mathrm{in}-\theta_\mathrm{out}}{3AR} + O\left(\frac{1}{AR^2}, (\theta_\mathrm{in}-\theta_\mathrm{out})^2\right) = 0, \tag{6}$$

which implies that for $0 < \theta_\mathrm{in} - \theta_\mathrm{out} \ll 1$ and $AR \gg 1$ the term inversely proportional to AR and the following O terms can be dropped, thus, leading to the relation

$$\theta_\mathrm{out} - \theta_\mathrm{in} \approx \frac{\sin 2\theta_\mathrm{e}}{6AR}. \tag{7}$$

Then, the capillary force from the difference in contact angles can be approximated by,

$$\boldsymbol{f}_\mathrm{cap} = \gamma(\cos\theta_\mathrm{in} - \cos\theta_\mathrm{out})\,\hat{\boldsymbol{r}} \approx -\frac{\gamma}{3AR}\cos\theta_\mathrm{e}\sin^2\theta_\mathrm{e}\,\hat{\boldsymbol{r}}, \tag{8}$$

where Eq. (7) was used in the approximation. Under a similar argument when using Eq. (1), dissipative forces can be expressed as,

$$\boldsymbol{f}_\mathrm{dis} = -\frac{\mathrm{d}R_\mathrm{m}}{\mathrm{d}t}\frac{\eta}{\sin\theta_e}\log\frac{\bar{w}}{\xi}\,\hat{\boldsymbol{r}}. \tag{9}$$

Then, the balance of forces, $f_{\text{cap}} + f_{\text{dis}} = 0$, result in the instantaneous velocity to collapse,

$$\frac{\mathrm{d}R_{\mathrm{m}}}{\mathrm{d}t} = -\frac{\gamma \sin\theta_{\mathrm{e}} \cos\theta_{\mathrm{e}}}{3\eta AR \log(w/\xi)} \sim -\frac{U^*}{3AR}. \tag{10}$$

We can give an estimate of the timescale for the radial collapse after integrating Eq. (10) in time, that is,

$$t_{n=0} \sim \frac{3R_{\mathrm{m}}AR}{U^*}. \tag{11}$$

In our experiments, $R_{\mathrm{m}} \approx 2\,\mathrm{mm}$, and a wide liquid filament ($AR \approx 2$), the radial collapse timescale evaluates to $t_{n=0} \sim 2.4\,\mathrm{s}$.

According to Brochard-Wyart and Redon [3], the P-R growthrate of a wavelength λ for a sessile liquid stripe is given by

$$\omega_{\mathrm{PR}}(\lambda) = \frac{\gamma\theta_{\mathrm{e}}^3}{3\eta \log(\bar{w}/\xi)} \cdot \frac{2\pi/\lambda}{1 + (\lambda/\pi\bar{w})^2} \left(\frac{\lambda}{\pi\bar{w}} - \tanh\frac{\pi\bar{w}}{\lambda}\right). \tag{12}$$

This expression has a maximum at $\lambda_c \approx 4\pi\bar{w}/3$, therefore,

$$\max\omega_{\mathrm{PR}} \approx \frac{3U^*}{50\bar{w}}\left(4 - 3\tanh\frac{3}{4}\right) \approx \frac{U^*}{8\bar{w}}. \tag{13}$$

We use this result to give an estimate of the timescale for the mode of maximum growthrate of the toroidal liquid filament,

$$t_{n^*} = \frac{1}{\max\omega_{\mathrm{PR}}} \approx \frac{8\bar{w}}{U^*} \sim \frac{8R_{\mathrm{m}}}{U^*AR}. \tag{14}$$

The characteristic time for the fastest mode is given by $t_{n^*} \sim 0.53\,\mathrm{s}$ for a slim toroidal liquid filament ($AR \approx 6$).

Mathematical model of the toroidal liquid filament

Governing equations

We model the stability and dynamics of the toroidal liquid filament around the equilibrium state based on the long wavelength film equation [4] in a similar fashion as in ref. [2],

$$\partial_t h - \frac{\gamma}{3\eta}\nabla\cdot\left[h^2(h + 3\ell)\nabla\hat{\kappa}\{h\}\right] = 0, \tag{15}$$

where h is the local thickness of the film, η is the viscosity of the liquid and γ is the surface tension of the liquid-gas interface, and $\hat{\kappa}$ is the mean curvature operator acting on h, i.e., $\hat{\kappa}\{h\} = \nabla\cdot\hat{\mathbf{n}}$, $\hat{\mathbf{n}}$ is the unitary normal vector to the liquid-gas interface.

In Eq. (15), it is assumed that the velocity field inside the toroidal liquid filament follows a Poiseuille profile subject to the boundary conditions at the solid surface:

$$0 = \hat{\mathbf{n}}_{\mathrm{s}}\cdot\mathbf{u}(\mathbf{x}_{\mathrm{s}}), \tag{16}$$

$$0 = \left(\mathbf{I} - \hat{\mathbf{n}}_{\mathrm{s}}\hat{\mathbf{n}}_{\mathrm{s}}^T\right)\mathbf{u}(\mathbf{x}_{\mathrm{s}}) - \ell\,\hat{\mathbf{n}}_{\mathrm{s}}\left[\nabla\mathbf{u}(\mathbf{x}_{\mathrm{s}}) + \nabla\mathbf{u}(\mathbf{x}_{\mathrm{s}})^T\right](\mathbf{I} - \hat{\mathbf{n}}_{\mathrm{s}}\hat{\mathbf{n}}_{\mathrm{s}}^T), \tag{17}$$

where $\hat{\mathbf{n}}_{\mathrm{s}}$ represents the normal unitary vector to the surface, $\mathbf{x}_{\mathrm{s}}$ is the position vector on the solid surface and the superscript T transposes the column vector into a row vector [5]. Eq. (16) corresponds to the impenetrability condition which restricts the motion of the fluid into the solid, while Eq. (17) is know as the Navier-slip condition that allows contact lines to move [4].

We are interested in modelling interfaces with contact angles close to 90°, harnessing the symmetry of the system, we parametrise the shape of the surface in toroidal coordinates. The translation from the toroidal coordinate system to the Cartesian, $(x, y, z) \leftrightarrow (\sigma, \tau, \psi)$, is defined by

$$\left(x, y, z\right) \equiv \frac{a}{\cosh\sigma - \cos\tau}\left(\sinh\sigma\cos\psi, \sinh\sigma\sin\psi, \sin\tau\right), \tag{18}$$

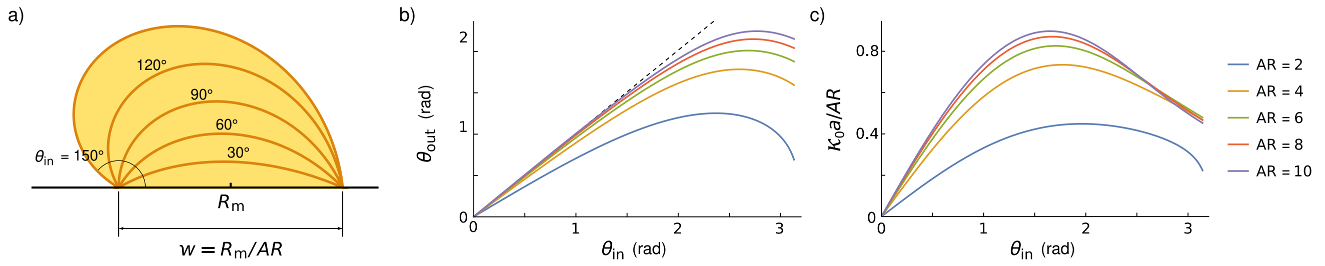

Supplementary Figure S2: Solutions to the constant curvature for fixed end points. (a) Shape of the interface for a toroidal liquid filament of aspect ratio $AR = 3$. (b) Relation between the contact angles at different values of AR (solid curves), contrasted by the curve $\theta_{out} = \theta_{in}$ (dashed line). (c) Dependence of the curvature on the inner contact angle.

where a is the radius of the reference circle, $\sigma \geq 0$ is the logarithm of the distances to the reference circle, that is, the locus $\sigma =$ const., forms regular tori. $\tau \in [0, \pi]$, is the angle of a spherical bowl that opens from the $z = 0$ plane at the reference circle, and $\psi \in [0, 2\pi]$ is the azimuthal coordinate [6].

We parametrise the liquid-gas interface by equating $\sigma = S(\tau, \psi, t)$. Due to the change in the coordinates system, Eq. (15) takes the form

$$\frac{a \cosh S}{\sinh^2 S} \partial_t S + \frac{\gamma a^3}{3\eta} \nabla \cdot \left[h^2 \left(h + 3\frac{\ell}{a} \right) \nabla \hat{\kappa}\{S\} \right] = 0, \tag{19}$$

where we identify $h = z \circ S = \sin\tau/(\cosh S - \cos\tau)$ and we compute $\partial_t h = \partial_S h\, \partial_t S$ with the approximation $\partial_S h \approx -a \cosh S/\sinh^2 S$ to avoid the singularities at the end points, $\tau = 0, \pi$.

To obtain the stability of toroidal liquid filaments around equilibrium, we assume that the form of the surface can be expressed by a sinusoidal perturbation with an exponential growth, $\cos n\psi\, e^{\omega t}$, about equilibrium,

$$S(\tau, \psi, t) = S_0(\tau) + \epsilon\, S_1(\tau) \cos n\psi\, e^{\omega t}, \tag{20}$$

where ϵ is the amplitude of the perturbation and n is the wave-mode of the perturbation and ω is the growthrate. We carry out a linear stability analysis where we substitute Eq. (20) in Eq. (19) and sort terms in powers of ϵ up to linear order.

Equilibrium solution

We begin by finding S_0, the equilibrium surface. For it to be a temporal equilibrium state, we require that the Laplace pressure is homogeneous within the liquid phase, and thus, solution to the equation $\hat{\kappa}\{S_0\} = \kappa_0$ for a constant κ_0, i.e.,

$$\frac{(1 + S_0'^2)^2}{\sinh S_0} \left[\cos\tau \cosh S_0 + \sinh^2 S_0 - 2 \sin\tau \sinh S_0 S_0' - 1 \right] + (\cosh S_0 - \cos\tau) S_0'' = a\kappa_0 (1 + S_0'^2)^{3/2}, \tag{21}$$

where the primed notation represents differentiation with respect to τ. Eq. (21) is a second order differential equation, and thus, to give closure and unity to the solutions, two boundary conditions are required, namely

$$S_0(0) = S_0(\pi) = \log\left[AR + \sqrt{4AR^2 - 1} \right]. \tag{22}$$

For a given value of κ_0, both contact angles are specified by the relations

$$S_0'(0) = \frac{1}{\tan\theta_{out}}, \qquad \text{and} \qquad S_0'(\pi) = -\frac{1}{\tan\theta_{in}}, \tag{23}$$

where θ_{in} and θ_{out} are the inner and outer contact angles, respectively (see Fig. S2a). Therefore, we are able to specify the inner or the outer contact angles by finding a value of κ_0 and the opposing contact angle as shown in Fig. S2b,c.

This boundary value problem was solved numerically using the Python™ function `solve_bvp` from the module `scipy.integrate` [7] with 512 collocation points.

Linear Stability Analysis

The next equation of motion corresponds to linear order in ϵ, this is,

$$\Omega \frac{\cosh S_0}{\sinh^2 S_0} S_1 \cos n\psi e^{\omega t} + a\nabla \cdot \left[h_0^2 \left(h_0 + 3\frac{\ell}{a} \right) \nabla \kappa_1(\tau, \psi) \right] = 0, \tag{24}$$

where

$$\Omega := \frac{3\eta\omega}{\gamma a} \tag{25}$$

is the dimensionless growthrate, $h_0 = \sin\tau/(\cosh S_0 - \cos\tau)$, and κ_1 is such that,

$$\hat{\kappa}\{S\} = \kappa_0 + \epsilon\kappa_1 + O(\epsilon^2), \tag{26}$$

We can express the variation of the curvature in terms of the normal displacement field, ζ, according to Brinkmann, *et al.* (2004) [8, 11],

$$\kappa_1 = \hat{\kappa}_1\{S\} = \left[\nabla^2 + \kappa_0^2 - 2g_0(\tau) \right] \zeta(\tau) \cos n\psi \, e^{\omega t}, \tag{27}$$

where g_0 is the Gaussian curvature of the surface S_0. S_1 and ζ are related by the expression

$$S_1(\tau) = \frac{\cosh S_0 - \cos\tau}{a\left[1 + S_0'^2\right]^{1/2}} \zeta(\tau). \tag{28}$$

Eq. (28), together with Eq. (27), are substituted into Eq. (24) to form an eigenvalue equation, where Ω and $\zeta(\tau)$ are the eigenvalue and eigenfunction, respectively.

The differential operators, take the form,

$$\nabla f = \sum_{i,j} \mathsf{g}^{ij} \partial_i f \, \hat{\mathbf{e}}_j, \tag{29}$$

$$\nabla \cdot \mathbf{A} = \sum_i |\mathsf{g}|^{-1/2} \partial_i \left(|\mathsf{g}|^{1/2} A_i \right) \tag{30}$$

and, therefore, the Laplace-Beltrami operator is expressed as,

$$\nabla^2 f = \sum_{i,j} |\mathsf{g}|^{-1/2} \partial_i \left(|\mathsf{g}|^{1/2} \mathsf{g}^{ij} \partial_j f \right), \tag{31}$$

for some scalar field, f, and vector field, $\mathbf{A}$. Here, g_{ij}, with lower indices, is the induced metric tensor on the surface S_0,

$$\mathsf{g}_{ij} = \frac{a^2}{(\cosh S_0 - \cos\tau)^2} \begin{pmatrix} 1 + S_0'^2 & 0 \\ 0 & \sinh^2 S_0 \end{pmatrix}. \tag{32}$$

Therefore, g^{ij}, with upper indices, is the inverse of the metric tensor, and $|\mathsf{g}|$ is its determinant,

$$|\mathsf{g}| = \det \mathsf{g}^{ij} = \frac{(\cosh S_0 - \cos\tau)^4}{a^4(1 + S_0'^2)\sinh^2 S_0}. \tag{33}$$

To give closure to the system, we establish the boundary conditions for the eigenvalue problem. We use the same boundary conditions of Eqs. (16) and (17). By taking the derivative with respect to time we are able to calculate the velocity at the liquid-gas interface,

$$u_\sigma = \partial_t S = \epsilon\omega S_1 \cos n\psi \, e^{\omega t}. \tag{34}$$

We use Eq. (34) at the boundary points, $\tau = 0, \pi$, in Eqs. (16) and (17) of the initial thin-film equation for consistency. This boundary conditions are implemented for consistency, they allow the motion of the contact line while at the cost of altering the contact angle dynamically. In this way, the boundary conditions recast into

$$0 = \left(\frac{\sinh S_0 S_0' \mp a/\ell}{\cosh S_0 \mp 1} - \frac{S_0' S_0''}{1 + S_0'^2} \right) \zeta + \zeta', \tag{35}$$

where the first choice of signs correspond to $\tau = 0$ and the second to $\tau = \pi$.

To solve the eigenvalue equation, we use pseudo-spectral methods [9]. We implement a Gauss-Lobatto grid of 64 points and the boundary conditions are prescribed by setting a linear combination of Chebyshev polynomials, T_i, that define the base functions, $\phi_i(\tau)$, for the numerical method,

$$\phi_i(\tau) \equiv T_i(2\tau/\pi - 1) + \alpha_i T_{i+1}(2\tau/\pi - 1) + \beta_i T_{i+2}(2\tau/\pi - 1), \tag{36}$$

so that the solution is expressed as

$$\zeta(\tau) = \sum_{i=0}^{N} b_i \phi_i(\tau), \tag{37}$$

and b_i are unknown coefficients that satisfy the eigensystem problem.

The master curve and maximum growth rate

From the experimental results (see Fig. 3a of the main text), it is observed that the growth rate behaves as a forth degree polynomial with respect to n. This polynomial is of the form $a_0 + a_2 n^2 - a_4 n^4$, since the odd powers must vanish as the system is invariant upon a change in its sign ($n \longrightarrow -n$). Moreover, at high values of AR and low values of ℓ, the inner contact line shows low mobility, therefore, the growth rate that corresponds to $n = 0$ is negligible.

By carrying out numerical analysis over ranges of contact angles ($\theta_{\text{out}} \in [50°, 90°]$) and values of the mobility ($\ell/R_{\text{m}} \in [0.05, 0.25]$) containing our experimental parameters ($\theta_{\text{out}} \approx 72°$, $\ell/R_{\text{m}} \approx 0.16$), it was found that with a parameter transformation, the dependence can be collapsed into a master curve. The transformation is

$$N := \frac{n}{AR - \beta}, \tag{38}$$

where

$$\beta = \beta(\theta_{\text{out}}, \ell) \approx \exp\left\{[\alpha_1 \sin(2\theta_{\text{out}}) + \alpha_2][\log(\ell/a) + 1] + \alpha_3\right\}, \tag{39}$$

and the parameters α_i, $i = 1, 2, 3$ were found via curve fitting: $\alpha_1 = -0.913$, $\alpha_2 = -0.187$, and $\alpha_3 = -0.733$, and an overall deviation of ±2.3%.

By using Eq. (38), the dependence of all parameters in the growth rate is condensed into the master curve following master curve,

$$\Omega(n, AR, \theta_{\text{out}}, \ell) \underset{\sim}{\propto} 2\, N_{\max}^2\, N^2 - N^4, \tag{40}$$

where $N_{\max}$ corresponds to the N of maximum growth rate ($N_{\max} = \arg\max \Omega(N)$).

Once again, the expression for $N_{\max}$ is found by proposing a curve with free parameters to fit,

$$N_{\max} := \frac{n_{\max}}{AR - \beta} \approx \frac{\pi}{2}\left\{1 + \alpha_4\left[\frac{a}{\ell} - \cos 2\theta_{\text{out}} \log\frac{\ell}{a}\right] + \alpha_5\left[\log\frac{\ell}{a} + \sin 2\theta_{\text{out}}\right] + \alpha_6 \frac{\cos 2\theta_{\text{out}}}{\log(\ell/a)}\right\}, \tag{41}$$

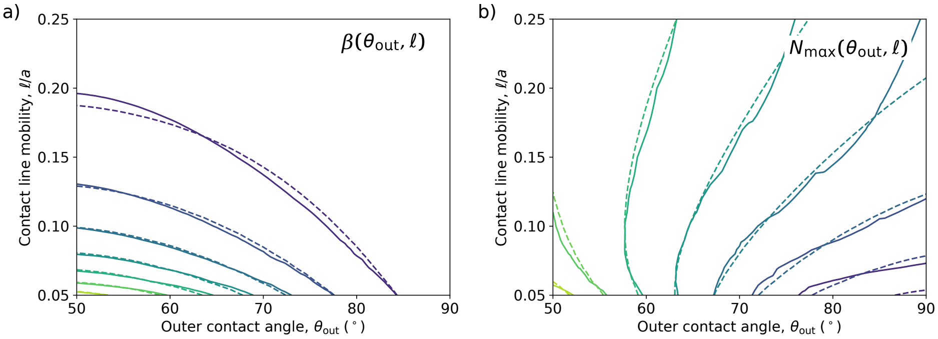

Supplementary Figure S3: Contour plots of the numerical values (solid lines) and fitting functions (dashed lines) for (a) σ and (b) $N_{\max}$ in the parameter region $\theta_{\text{out}} \in [50°, 90°]$ and $\ell/R_{\text{m}} \in [0.05, 0.25]$.

where the parameters that fit our numerical results are: $\alpha_4 = 0.014$, $\alpha_5 = 0.06$, and $\alpha_6 = 0.018$, with an average deviation of ±1.6% over the region of interest.

The numerical values for fitting the trial functions β and $N_{\max}$ in Eq. (39) and Eq. (41) can be obtained by extracting the slope and intercept of a straight line for the maximum growth rate at a given aspect ratio. This is done for each pair of parameters θ_{out} and ℓ. The accuracy of the fit for both functions is shown in Fig. S3 by contrasting the contour plots of the numerical results and the trial functions.

Voltage controlled retraction

In this section we explain the effect of a non-zero retracting voltage in the evolution of the Plateau-Rayleigh instability. For this, we measure the response of the contact angle and the width of the toroidal liquid filament for a given voltage, V, and we produce a simplified model for the effect on the mobility of the contact lines.

Based on the dielectrowetting equation [10], the contact angle is expected to vary as,

$$\cos\theta(V) = \cos\theta_{\text{out}} + \alpha V^2. \tag{42}$$

From the experiments (see Sup. Fig. S4(a)), we found $\theta_{\text{out}} = 72^\circ \pm 1^\circ$ and $\alpha = 1\times10^{-5} \pm 1.3\times10^{-6}\,\text{V}^2$. The variation of the aspect ratio, AR, can be synthesised by the expression

$$AR(V) = \min\{AR_0, \alpha_1 V + \alpha_2\}, \tag{43}$$

where AR_0, is the value at zero retraction voltage, and $\alpha_1 = -0.014\,\text{V}^{-1}$ and $\alpha_2 = 7.8561$ were obtained from the measurements as shown in Sup. Fig. S4(b).

The effect of the mobility of the contact line was not measured directly, and therefore, we have produced a simplified model to account for its effect. In order to do this, we first examine the forces at the contact lines. During the retraction of the initial film, the liquid encounters the underlining structure of the electrodes which are concentric circle of alternating polarity. The intensity of the electric field becomes periodic, with wavelength λ, in the radial coordinate, R. Since the dielectrophoretic forces are independent of the polarity, we model the intensity of the electric field proportional to $\cos^2(2\pi R/\lambda)$ (see Sup. Fig. S5a). This leads to model the dielectrophoretic force for dewetting along the perpendicular direction to the electrodes, $f_{\text{el}}^{\perp}$, as

$$f_{\text{el}}^{\perp}(V,R) = f_{\text{el}}(V)\cos^2(2\pi R/\lambda) = \frac{\Delta\varepsilon}{2\delta}V^2\cos^2(2\pi R/\lambda) = \alpha\gamma V^2\cos^2(2\pi R/\lambda), \tag{44}$$

where $\Delta\varepsilon$ is the difference in dielectric permittivity between the dielectric liquid and the surrounding gas, δ is the penetration depth which models the effective range of the electrostatic field.

The force in Eq. (44) is compensated by the surface tension forces that also act on the contact line. At equilibrium the forces must balance, therefore, we write, for the outer contact line,

$$f_{\text{res}}(V,R) = \gamma\cos\theta_{\text{out}} - \gamma\cos\theta(V) + f_{\text{el}}(V)\cos^2(2\pi R/\lambda) = 0, \tag{45}$$

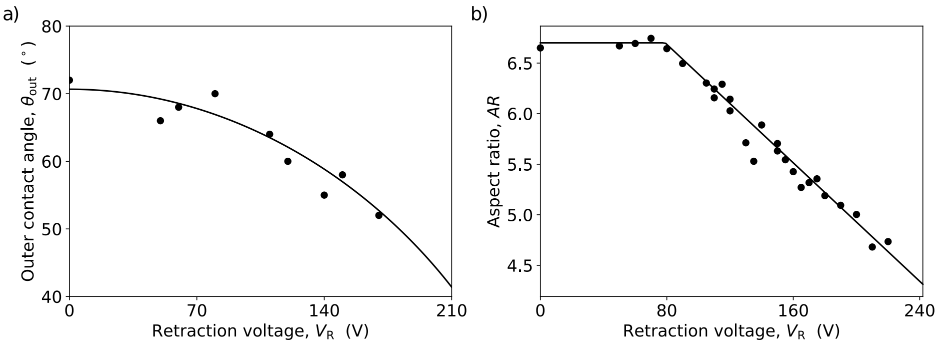

Supplementary Figure S4: Variation of the outer contact angle, θ_{out} (a), and aspect ratio, AR (b), with respect to the retraction voltage, V_{R}. The experimental values (scatter plot) are fitted (solid the) by Eqs. (42) and (43).

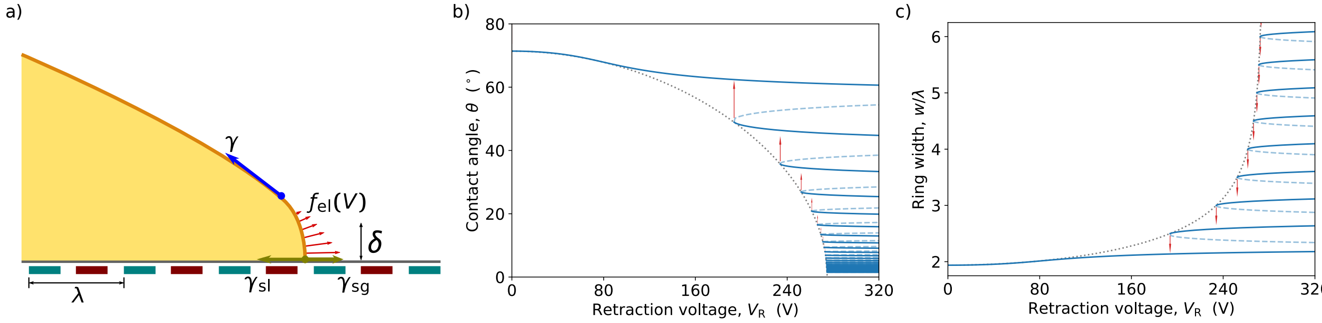

Supplementary Figure S5: Simplified model of a receding contact line when electric fields are present. (a) Schematic representation of the forces acting at the contact line due to a finite retraction voltage. The electrodes on the solid surface (line segments of alternating colour) are perpendicular to the motion of the contact line. The competing forces are represented by arrows: the surface tension of the liquid-gas interface (blue), the electrostatic force (red) and the difference of the solid surface tensions (olive). Plots of the value of the contact angle (b) and width of the toroidal liquid filament (c) according to the model. The solid lines represent stable states where the interface becomes effectively pinned, in contrast to the dashed lines that are unstable. The vertical arrows represent sudden jumps in the equilibrium configuration as the voltage is reduced. The grey dotted line corresponds to the case of dewetting in parallel.

where $f_{\rm res}$ is the residual force, and for the inner contact line, a similar expression replacing $\theta_{\rm out} \longrightarrow \theta_{\rm in}$ and inverting signs. Eq. (45) implies that the contact lines are found at concentric rings of radius R^*, such that $f_{\rm res}(V, R^*) = 0$, i.e., the roots of the residual force.

For simplicity, we shall assume that the cross-sectional area is preserved. This assumption is justified from the conservation of volume which approximately preserves the cross-sectional area if the central collapse is negligible. Then, we consider toroidal liquid filaments of circular cross-section of area, A, and width, $w := R_{\rm o} - R_{\rm i}$ given by the two roots of the residual force. Then,

$$A(V, R) := \left[\frac{w}{\sin\theta(V)}\right]^2 [\theta(V) - \sin\theta(V)\, \cos\theta(V)] = \text{const.} \tag{46}$$

Eqs. (45) and (46) give closure to the system.

We proceed to assess the stability of the position of the contact lines in order to determine the effect of perturbations. This can be done by considering a small change, δR, in the position of the contact lines with respect to its equilibrium value, thus, the forces at the contact line are given by $f_{\rm res}(V, R^* + \delta R) \approx \partial_R f_{\rm res}(V, R^*)\delta R$. If, $\partial_R f_{\rm res} > 0$, then, the force increases in the direction of the displacement, therefore, classifying R^* as an unstable position, whereas $\partial_R f_{\rm res} < 0$ classifies it as stable. This is shown in Sup. Fig. S5(b, c).

Provided the exponential evolution of the Plateau-Rayleigh instability, δR is proportional to the velocity of the contact lines $v_{\rm cl}$, in more detail, $\delta R = \int v_{\rm cl} {\rm d}t \propto \int e^{\omega t} {\rm d}t \propto v_{\rm cl}$. Therefore, the quantity $b\partial_R f_{\rm res}(V, R^*) v_{cl}$, where b is a constant of proportionality, is also equivalent to the restoring force. This implies that, during the Plateau-Rayleigh regime, for a voltage controlled retraction, an additional term is present in boundary condition,

$$0 = \left[\frac{\eta}{\ell} + b\partial_R f_{\rm res}\right] (\mathbf{I} - \hat{\mathbf{n}}_{\rm s}\hat{\mathbf{n}}_{\rm s}^T)\, \mathbf{u}(\mathbf{x}_{\rm s}) - \eta\, \hat{\mathbf{n}}_{\rm s} \left[\nabla\mathbf{u}(\mathbf{x}_{\rm s}) + \nabla\mathbf{u}(\mathbf{x}_{\rm s})^T\right] (\mathbf{I} - \hat{\mathbf{n}}_{\rm s}\hat{\mathbf{n}}_{\rm s}^T). \tag{47}$$

The terms in the first squared brackets are the mobility of the contact line as detailed in Eq. (17), and the resistance to motion due to the electric forces on the interface. From this term in brackets, we can simplify Eq. (47) by defining an effective mobility of the contact lines, $\ell_{\rm eff}$,

$$\frac{1}{\ell_{\rm eff}(V)} = \frac{1}{\ell} + \frac{b}{\eta}\partial_R f_{\rm res}(V, R^*). \tag{48}$$

Furthermore, from Eq. (45) we have $\partial_R f_{\rm res} = -(2\pi/\lambda)\, f_{\rm el}(V) \sin(4\pi R/\lambda)$, and thus, $\partial_R f_{\rm res} \sim \alpha\gamma V^2$. Then, as $\lambda \ll R^*$, we assign

$$\frac{1}{\ell_{\rm eff}(V)} \approx \frac{1}{\ell} + \alpha b' V^2. \tag{49}$$

Eq. (49) implies that, as the retraction voltage increases, the forces that produce the spreading of the liquid become stronger. Due to the variation in the intensity of the electric field, these manifest as pinning points. At low voltages, $\ell_{\text{eff}} \to \ell$, whereas at high voltages, the dielectrophoretic forces pin the contact line thus asymptotically reducing the mobility to zero.

References

[1] Snoeijer, J. H. (2006) Free-surface flows with large slopes: Beyond lubrication theory. Physics of Fluids 18(2):021701.

[2] González AG, Diez JA, Kondic L (2013) Stability of a liquid ring on a substrate. Journal of Fluid Mechanics 718:246–279.

[3] Brochard-Wyart F, Redon, C (1992) Dynamics of liquid rim instabilities. Langmuir 9(8):2324–2329.

[4] Oron A, Davis SH, Bankoff SG. (1997) Long-scale evolution of thin liquid films. Rev. Mod. Phys. 69(3):931–980.

[5] Einzel D, Panzer P, Liu M. (1990) Boundary condition for fluid flow: Curved or rough surfaces. Phys. Rev. Lett. 64(19):2269–2272.

[6] Moon P, Spencer DE (1971) Field Theory Handbook Including Coordinate Systems Differential Equations and Their Solutions. (Springer-Verlag), 2 edition.

[7] Jones E, Oliphant T, Peterson P, , et al. (2001–) SciPy: Open source scientific tools for Python.

[8] Brinkmann M, Kierfeld J, Lipowsky R (2004) A general stability criterion for droplets on structured substrates. Journal of Physics A: Mathematical and General 37(48):11547–11573.

[9] Boyd JP (2001) Chebyshev and Fourier spectral methods. (Courier Corporation).

[10] McHale G, Brown CV, Newton MI, Wells GG, Sampara N. (2011) Dielectrowetting driven spreading of droplets. Physical Review Letters, 107(18):186101.

[11] Bostwick, J. B. and Steen, P. H. (2018) Static rivulet instabilities: varicose and sinuous modes. Journal of Fluid Mechanics, 837:819–838.
